# Supplementary material for: The ecology and evolution of sub-exponential replicators
Source: PLoS Comput Biol. 2026 May 8;22(5):e1014247. doi: 10.1371/journal.pcbi.1014247 (PMC13155683; doi:10.1371/journal.pcbi.1014247)
Supplement: S1 Appendix — (PDF) [file pcbi.1014247.s001.pdf]

Supporting information

for

The ecology and evolution of sub-exponential replicators

Bianka Kovács, György Barabás, Géza Meszéna,
Eörs Szathmáry, András Szilágyi

Contents

Appendix A: Detailed analysis of the mass-regulated model **2**
A.1 Regulation on the total replicator concentration, normalized production 2
A.2 Matrix-vector formalism, growth rates 3
A.3 Coexistence of different replicator species 4
A.3.1 Coexistence of S-species 4
A.3.2 Coexistence of S-species and one E-species 5
A.4 Invasion into a system being at equilibrium 7
A.5 The production of the system cannot decrease after successful invasion 7
A.6 The largest coexisting subset of a library of replicator species 9
A.6.1 Evolutionary stability: a serial invasion of species 10
A.7 Ecological stability of coexisting replicators in the mass-regulated model 10
A.7.1 The impact of changing the parameters of a single species on the equilibrium of a purely sub-exponential system 12
A.7.2 The impact of changing the parameters of a single species on the equilibrium of a system containing an E-species 14
A.7.3 The critical parameter values that lead to the first extinction in a given coalition 15
A.8 Parabolic dynamics of a single species at high total concentration 16
A.9 Diversity-maintaining ability in the abundance of resources 17

Appendix B: Resource-regulated model **20**
B.1 Coexistence of different replicator species 21
B.1.1 Coexistence of S-species 22
B.1.2 Coexistence of S-species and one E-species 23
B.2 Invasion of a new species into an established community 24
B.3 The resource concentration cannot increase after successful invasion 27
B.4 The largest coexisting subset of a library of replicator species 28
B.5 Ecological stability of coexisting replicators in the chemostat 30
B.5.1 Changing the parameters of a single species in a purely sub-exponential system 30
B.5.2 Changing the parameters of a single species in a system that contains an E-species 32
B.5.3 Changing the external parameters 32
B.5.4 The 'critical' parameter values that lead to the first extinction in a given coalition 33
B.6 The effect of changes in the external parameters ρ and φ on the composition of coexisting species 34
B.6.1 The effect of changing ρ 34
B.6.2 The effect of changing φ 36

Appendix C: The phenomenological model **38**
C.1 Dynamics of the system 38
C.2 Coexistence of different replicator species in the phenomenological model 38
C.2.1 Coexistence of S-species 38
C.2.2 Coexistence of S-species and one E-species 39
C.3 Invasion analysis in the phenomenological model 41
C.3.1 Invasion of an S-species into an established sub-exponential community . . . 41

C.3.2 Invasion of an E-species into an established sub-exponential community . . . 41
C.3.3 Invasion of an S-species into an established exponential community 42
C.3.4 Invasion of an E-species into an established exponential community 42

References **43**

Appendix A: Detailed analysis of the mass-regulated model

A.1 Regulation on the total replicator concentration, normalized production

The dynamics of the association–dissociation–replication process can be described for a replicator species i by the following set of differential equations:

$$\begin{aligned} \dot{x}_i &= -2a_i x_i^2 + 2b_i y_i - rc_i x_i - \varphi x_i, \\ \dot{y}_i &= a_i x_i^2 - b_i y_i + rc_i x_i - \varphi y_i, \end{aligned} \tag{A.1}$$

where the dot denotes differentiation with respect to time (so $\dot{x}_i = \mathrm{d}x_i/\mathrm{d}t$). Otherwise, see Eq. 2 and the corresponding section in the main text for notations.

Let us introduce the total concentration of species i as $\mu_i = x_i + 2y_i$ and the total replicator concentration of the whole system as $\mu = \sum_{i=1}^{N} \mu_i = \sum_{i=1}^{N} x_i + 2y_i$. By defining the system's relative outflow (i.e., its total outflow divided by μ) with the normalized production written as

$$\varphi = \frac{r}{m} \sum_{j=1}^{N} c_j x_j \tag{A.2}$$

(cf. Eq. 3 in the main text), the time derivative of the total replicator concentration can be computed as

$$\dot{\mu} = \sum_{i=1}^{N} (\dot{x}_i + 2\dot{y}_i) = r \sum_{i=1}^{N} c_i x_i - \frac{r}{m} \sum_{j=1}^{N} c_j x_j \sum_{i=1}^{N} (x_i + 2y_i) = r \sum_{j=1}^{N} c_j x_j \left[1 - \frac{\mu}{m}\right]. \tag{A.3}$$

Consequently, the regulation implemented by Eq. A.2 keeps the equilibrium total replicator concentration $\hat{\mu}$ at m via a logistic-type regulation. Note that the dynamics of the total concentration of species i is

$$\dot{\mu}_i = rc_i x_i - \varphi \mu_i. \tag{A.4}$$

From Eq. A.1, the growth rate of species i (i.e., its per capita production) is $\frac{rc_i x_i}{\mu_i}$, while the growth rate of the system is $\frac{r \sum_{j=1}^{N} c_j x_j}{\mu}$. At equilibrium, the growth rate of all species and also the growth rate of the whole system must be equal to the system's relative outflow to keep the total concentration fixed, yielding the equation

$$\hat{\varphi} = \frac{r \sum_{j=1}^{N} c_j \hat{x}_j}{m} = \frac{rc_i \hat{x}_i}{\hat{\mu}_i} \tag{A.5}$$

for the equilibrium value of the normalized production φ. Note that, using the definition $\hat{\mu}_i = \hat{x}_i + 2\hat{y}_i$, $\hat{\varphi}$ can also be expressed with the equilibrium ratio of double- vs. single-stranded replicators of any species, namely as

$$\hat{\varphi} = \frac{rc_i}{1 + 2\frac{\hat{y}_i}{\hat{x}_i}}. \tag{A.6}$$

A.2 Matrix-vector formalism, growth rates

A population of template-directed replicators is a structured one, as it consist of single- and double-stranded replicators. Dynamics of such populations can be described by a matrix, or more generally by a linear operator [1, 2]. Here, the dynamics of single- and double-stranded replicators is determined by a population projection matrix $\mathbf{M}$, while φ defines the (non-selective) flux of the outflow. Using these, Eq. A.1 can be written in matrix-vector form as

$$\frac{\mathrm{d}}{\mathrm{d}t}\begin{pmatrix} x_i \\ y_i \end{pmatrix} = (\mathbf{M} - \varphi\mathbf{I})\begin{pmatrix} x_i \\ y_i \end{pmatrix}, \tag{A.7}$$

where

$$\mathbf{M} = \begin{bmatrix} -2a_ix_i - rc_i & 2b_i \\ a_ix_i + rc_i & -b_i \end{bmatrix} \tag{A.8}$$

and $\mathbf{I}$ denotes the identity matrix.

Consider now an equilibrium solution of Eq. A.7. Because of the equilibrium assumption, the state vector $\begin{pmatrix} x_i \\ y_i \end{pmatrix}$ is constant, and so is the matrix $\mathbf{M}$. Then, the state vector should be an eigenvector of matrix $\mathbf{M}$ with eigenvalue φ. Accordingly, the equilibrium can be seen, as a balance between an exponential growth and an outflow, both of them with rate φ.

Stability of the equilibrium requires the eigenvalue in question, which is equal to φ, being the leading eigenvalue. Otherwise, the fixed point were unstable against a perturbation in the eigendirection corresponding to an eigenvalue larger than φ. The leading eigenvalue of $\mathbf{M}$ given by

$$\Lambda_i(x_i) = \frac{-(2a_ix_i + b_i + rc_i) + \sqrt{(2a_ix_i + b_i + rc_i)^2 + 4rb_ic_i}}{2} \tag{A.9}$$

is the growth rate of species i (corresponding to its per capita production rc_ix_i/μ_i) at the single-stranded replicator concentration x_i.

It is a decreasing function of x_i since

$$\frac{\mathrm{d}\Lambda_i(x_i)}{\mathrm{d}x_i} = \left(-1 + \frac{2a_ix_i + b_i + rc_i}{\sqrt{(2a_ix_i + b_i + rc_i)^2 + 4rb_ic_i}}\right)a_i < 0. \tag{A.10}$$

The maximal value of the growth rate Λ_i, corresponding to $x_i \approx 0$, is the intrinsic growth rate:

$$\lambda_i = \Lambda_i(0) = \frac{-(b_i + c_ir) + \sqrt{(b_i + c_ir)^2 + 4b_ic_ir}}{2} \geq \Lambda_i(x_i). \tag{A.11}$$

The concentration-dependence of the growth rate Λ_i is scaled by a_i in Eq. A.10. The case $a_i = 0$ (no pairing) corresponds to exponential replicators, where the growth rate remains λ_i at any x_i concentration. The smaller the a_i, the longer the growth remains near exponential.

Based on the eigenvector corresponding to the eigenvalue λ_i, the ratio of double- vs. single-stranded replicators at $x_i \approx 0$ or at $a_i = 0$ can be written as

$$\frac{y_i}{x_i} = \frac{c_ir - \lambda_i}{2\lambda_i}. \tag{A.12}$$

Note that from the equilibrium concentration formulas, the ratio $\frac{\hat{y}_i}{\hat{x}_i} = \frac{c_ir - \hat{\varphi}}{2\hat{\varphi}}$ arises for any species i (both sub-exponential and exponential, cf. Eqs. A.15 and A.26), which is entirely in line with

the expectation that during the spreading of a new E-species from an infinitesimally small initial concentration, the ratio of its double- vs. single-stranded replicators does not change (as an E-species sets the normalized production exactly to its own intrinsic growth rate, see Fig. 1 in the main text), while an S-species (setting $\hat{\varphi}$ to a value lower than its own intrinsic growth rate, see Fig. 1 in the main text) shows increasing ratio of double- vs. single-stranded replicators with its growth due to the intensification of its association of single strands. Taking into account Eq. A.10 too, it can be claimed that there is an intuitively clear connection between three variables: a higher total concentration of a given S-species leads a lower ratio of its single-stranded replicators, resulting in a lower growth rate.

A.3 Coexistence of different replicator species

In this section we compute the steady state equilibrium of the system of Eq. 2 in the main text in the case of N S-species and in the case of a single E-species and N S-species.

A.3.1 Coexistence of S-species

In this subsection we derive the equilibrium concentrations for the system described in Eq. 2 in the main text. The steady state is defined by the following set of equations

$$\begin{aligned} -2a_i\hat{x}_i^2 + 2b_i\hat{y}_i - c_ir\hat{x}_i - \hat{x}_i\hat{\varphi} = 0 \\ a_i\hat{x}_i^2 - b_i\hat{y}_i + c_ir\hat{x}_i - \hat{y}_i\hat{\varphi} = 0, \end{aligned} \tag{A.13}$$

where we have used $\hat{\varphi} = \frac{r}{m}\sum_{j=1}^{N} c_j\hat{x}_j$, $i = 1 \ldots N$. Treating this as a known constant, the N independent equation pairs above can be easily solved, the result being

$$\begin{aligned} \hat{x}_i &= \frac{-\hat{\varphi}^2 - (b_i + c_ir)\hat{\varphi} + b_ic_ir}{2a_i\hat{\varphi}} = \frac{c_ir(b_i - \hat{\varphi}) - \hat{\varphi}(b_i + \hat{\varphi})}{2a_i\hat{\varphi}}, \\ \hat{y}_i &= \frac{\hat{\varphi}^3 + b_i\hat{\varphi}^2 - c_ir(c_ir + 2b_i)\hat{\varphi} + b_ic_i^2r^2}{4a_i\hat{\varphi}^2} = \frac{\left[c_ir(b_i - \hat{\varphi}) - \hat{\varphi}(b_i + \hat{\varphi})\right](c_ir - \hat{\varphi})}{4a_i\hat{\varphi}^2}. \end{aligned} \tag{A.14}$$

From this, the equilibrium ratio of double- vs. single-stranded replicators of an S-species i can be expressed as

$$\frac{\hat{y}_i}{\hat{x}_i} = \frac{c_ir - \hat{\varphi}}{2\hat{\varphi}}. \tag{A.15}$$

The equilibrium value of the normalized production can be expressed by $\hat{x}_i$'s:

$$\hat{\varphi} = \frac{r}{m}\sum_{j=1}^{N} c_j\hat{x}_j = \frac{r}{m}\sum_{j=1}^{N} c_j\left[\frac{-\hat{\varphi}^2 - (b_i + c_ir)\hat{\varphi} + b_ic_ir}{2a_i\hat{\varphi}}\right]. \tag{A.16}$$

After some rearrangement, we obtain the following quadratic equation

$$\left[2m + \sum_{j=1}^{N}\frac{c_jr}{a_j}\right]\hat{\varphi}^2 + \sum_{j=1}^{N}\frac{(b_j + c_jr)c_jr}{a_j}\hat{\varphi} - \sum_{j=1}^{N}\frac{b_jc_j^2r^2}{a_j} = 0. \tag{A.17}$$

or in a simple form:

$$(2m + \Theta_1)\hat{\varphi}^2 + \Theta_2\hat{\varphi} - \Theta_3 = 0, \tag{A.18}$$

where the notations

$$\Theta_1 = \sum_{j=1}^{N} \frac{c_j r}{a_j}, \quad \Theta_2 = \sum_{j=1}^{N} \frac{(b_j + c_j r) c_j r}{a_j}, \quad \Theta_3 = \sum_{j=1}^{N} \frac{b_j c_j^2 r^2}{a_j} \tag{A.19}$$

were used. Since all parameters are positive, the $(+, +, -)$ sign pattern guarantees that this equation has one positive root, which defines the equilibrium value of the normalized production:

$$\hat{\varphi} = \frac{-\Theta_2 + \sqrt{\Theta_2^2 + 4(2m + \Theta_1)\Theta_3}}{2(2m + \Theta_1)}. \tag{A.20}$$

Using this formula, the equilibrium concentrations can be calculated according to Eq. A.14.

As it can be seen directly from Eq. A.20, the equilibrium value of the normalized production is a decreasing function of m and behaves differently at low and high total concentrations: at low concentrations $\hat{\varphi}$ asymptotically tends to $\frac{-\Theta_2 + \sqrt{\Theta_2^2 + 4\Theta_1\Theta_3}}{2\Theta_1}$ with a gradually decreasing dependence on m, whereas at high total concentrations it is inversely proportional to the square root of the total concentration, i.e. $\hat{\varphi} \sim \frac{1}{\sqrt{m}}$. The qualitative explanation is straightforward. At higher total concentrations, the equilibrium concentrations of all species are higher. However, the increasing concentration of S-species increases their ratio of double- vs. single-stranded replicators, which, according to Eq. A.6, reduces their growth rate. Since at equilibrium, the normalized production is equal to the growth rate of each species, thus $\hat{\varphi}$ becomes smaller as m becomes larger. In the $m \to \infty$ limit, $\hat{\varphi} \to \sqrt{\frac{\Theta_3}{2m}}$.

According to Eq. A.20, the normalized production is an increasing function of the resource concentration. At low resource concentration (when r is small compared to $\frac{m}{\sum_{j=1}^{N} \frac{c_j}{a_j}}$), $\hat{\varphi}$ grows linearly with r, while at large r it saturates to $\frac{\sum_{j=1}^{N} \frac{b_j c_j^2}{a_j}}{\sum_{j=1}^{N} \frac{c_j^2}{a_j}}$ with a gradually decreasing dependence on r. Note that $\lim_{r \to 0} \hat{\varphi} = 0$: in the absence of resource, there is no production, and thus, no outflow from the system, the total replicator concentration remains at its initial value, but the association-dissociation equilibrium is reached.

A.3.2 Coexistence of S-species and one E-species

In this subsection, we extend the set of species by an E-species with index 0. (Note that more than one E-species cannot coexist, the best one excludes all other E-species.) For the E-species, the association rate is zero, i.e. $a_0 = 0$. The corresponding set of differential equations (Eq. 2 in the main text) is extended by the equations of the E-species:

$$\begin{aligned} \dot{x}_0 &= 2b_0 y - c_0 r x_0 - x_0 \varphi \\ \dot{y}_0 &= -b_0 y_0 + c_0 r x_0 - y_0 \varphi, \end{aligned} \tag{A.21}$$

where the relative outflow of the system is defined by the normalized production written as $\varphi = \frac{r}{m} \sum_{j=0}^{N} c_j x_j$.

The ratio of the concentration of double- and single-stranded replicators at equilibrium ($\dot{x}_0 = \dot{y}_0 = 0$) can be expressed from both equations: $\frac{\hat{y}_0}{\hat{x}_0} = \frac{c_0 r + \hat{\varphi}}{2b_0}$ and $\frac{\hat{y}_0}{\hat{x}_0} = \frac{c_0 r}{b_0 + \hat{\varphi}}$. Equating these two

and solving the resulting equation for $\hat{\varphi}$, we get the positive root as the equilibrium value of the normalized production:

$$\hat{\varphi} = \frac{-(b_0 + c_0 r) + \sqrt{b_0^2 + c_0^2 r^2 + 6 b_0 c_0 r}}{2} = \lambda_0, \tag{A.22}$$

which is the intrinsic growth rate of the E-species (i.e. the leading eigenvalue of the dynamical matrix $\mathbf{M}$ of Eq. A.8 at $a_0 = 0$). Consequently, the equilibrium production of the system is determined solely by the E-species.

Note that the fact that in the presence of an E-species the equilibrium value of the normalized production is determined by that species alone (Eq. A.22) is consistent with the normalized production for a system of only S-species (Eq. A.20). Assume a set of coexisting S-species ($a_i > 0$ for all i in the set) and for species k take the $a_k \to 0$ limit. In this case $\lim_{a_k \to 0} \Theta_1 = \frac{c_k r}{a_k}$, $\lim_{a_k \to 0} \Theta_2 = \frac{(b_k + c_k r) c_k r}{a_k}$, $\lim_{a_k \to 0} \Theta_3 = \frac{b_k c_k^2 r^2}{a_k}$ and with these

$$\lim_{a_k \to 0} \hat{\varphi} = \lambda_k. \tag{A.23}$$

Plugging $\hat{\varphi}$ into Eq. 2 in the main text, we get a set of equations determining the equilibrium concentration of each S-species separately:

$$\begin{aligned} \hat{x}_i &= \frac{-\hat{\varphi}^2 - (b_i + c_i r)\hat{\varphi} + b_i c_i r}{2 a_i \hat{\varphi}} = \frac{c_i r (b_i - \hat{\varphi}) - \hat{\varphi}(b_i + \hat{\varphi})}{2 a_i \hat{\varphi}}, \\ \hat{y}_i &= \frac{\hat{\varphi}^3 + b_i \hat{\varphi}^2 - c_i r (c_i r + 2 b_i)\hat{\varphi} + b_i c_i^2 r^2}{4 a_i \hat{\varphi}^2} = \frac{\left[c_i r (b_i - \hat{\varphi}) - \hat{\varphi}(b_i + \hat{\varphi})\right](c_i r - \hat{\varphi})}{4 a_i \hat{\varphi}^2}. \end{aligned} \tag{A.24}$$

I.e., the equilibrium concentrations of the S-species are the same as in Eq. A.14, but note that $\hat{\varphi}$ is defined here by Eq. A.22 instead of Eq. A.20.

The equilibrium concentration of the E-species can be computed by decomposing the equilibrium value of the normalized production as $\hat{\varphi} = \frac{r}{m} c_0 \hat{x}_0 + \frac{r}{m} \sum_{i=1}^{N} c_i \hat{x}_i$. This, using also the equilibrium ratio of double- and single-stranded replicators expressed from the equation $\dot{y}_0 = 0$ (based on Eq. A.21) as $\frac{\hat{y}_0}{\hat{x}_0} = \frac{c_0 r}{b_0 + \hat{\varphi}}$, yields

$$\begin{aligned} \hat{x}_0 &= \frac{m\hat{\varphi} - r \sum_{j=1}^{N} c_i \hat{x}_i}{c_0 r} \\ \hat{y}_0 &= \frac{m\hat{\varphi} - r \sum_{j=1}^{N} c_i \hat{x}_i}{\hat{\varphi} + b_0}. \end{aligned} \tag{A.25}$$

Using Eqs. A.22 and A.25, the ratio of the double- and single-stranded replicators of the E-species can be written in the same form as in the case of the S-species (cf. Eq. A.15), namely as

$$\frac{\hat{y}_0}{\hat{x}_0} = \frac{c_0 r - \hat{\varphi}}{2\hat{\varphi}}. \tag{A.26}$$

Note that the equilibrium ratio of double- vs. single-stranded replicators of an E-species depends solely on r, b_0 and c_0, while for a coexisting S-species i it depends on the replication rate c_i too.

A.4 Invasion into a system being at equilibrium

A new species can invade an established community if its boundary growth rate is positive, i.e. it can spread at low concentrations. Denoting the concentrations and the parameters of the invader by prime, the dynamics of its total concentration can be formulated based on Eqs. 2 and 3 in the main text as

$$\frac{\mathrm{d}\mu'}{\mathrm{d}t} = \frac{\mathrm{d}}{\mathrm{d}t}\left(x' + 2y'\right) = rc'x' - \left(\hat{\varphi}_{\mathrm{res}} + \frac{r}{m}c'x'\right)\mu' \approx c'rx' - \mu'\hat{\varphi}_{\mathrm{res}}, \tag{A.27}$$

where m and $\hat{\varphi}_{\mathrm{res}}$ denote the total replicator concentration and the normalized production of the resident community, respectively, and we assumed that the invader has low initial concentration and, therefore, low production compared to the resident system ($\mu' \ll m$, $c'rx' \ll m\hat{\varphi}_{\mathrm{res}}$). Note that the terms describing association and dissociation always drop out from Eq. A.27, thus it holds for both sub-exponential ($a' > 0$) and exponential ($a' = 0$) invaders.

The criterion for successful invasion is $\frac{\mathrm{d}\mu'}{\mathrm{d}t} > 0$, which can be written based on Eq. A.27 as

$$\hat{\varphi}_{\mathrm{res}} < \frac{c'rx'}{\mu'} \tag{A.28}$$

or, recognizing on the right side the invader's intrinsic growth rate $\lambda' = \frac{c'rx'}{\mu'}$, as

$$\hat{\varphi}_{\mathrm{res}} < \lambda'. \tag{A.29}$$

The obtained formula is easy to interpret: the new species can spread in an equilibrium system from an infinitesimally small initial concentration if its initial growth rate is greater than the growth rate of the resident species, which — because of the equilibrium — is equal for all the resident species to the per unit outflow of the system.

Note that at $\lambda_i = \hat{\varphi}$, $\frac{\mathrm{d}\mu_i}{\mathrm{d}t} = 0$, meaning that the concentration of species i does not change in time. Thus, if the normalized production of the system increases to the λ_i value of a resident S-species (corresponding to the possible largest growth rate of the given species), then this species remains in the system at a constant concentration of $\mu_i \approx 0$, which practically means that it dies out. Nonetheless, due to its concentration-independent growth rate, a resident E-species can maintain a finite concentration and survive even at $\hat{\varphi} = \lambda_0$ (if the total sub-exponential replicator concentration does not reach m at the normalized production value $\hat{\varphi}$). To sum up, as it is shown by Table A.1, the condition of spreading from an infinitesimally small concentration is given by Eq. A.29 for both S- and E-species, but resident exponential species can survive even at $\hat{\varphi} = \lambda_0$, while resident sub-exponential replicators remain in the system only if $\lambda_i > \hat{\varphi}$.

A.5 The production of the system cannot decrease after successful invasion

In the case of a purely sub-exponential system, the quadratic equation the positive root of which defines the normalized production at equilibrium can be written as follows (see Eq. A.17):

$$\left[2m + \sum_{j=1}^{N} \frac{c_j r}{a_j}\right]\hat{\varphi}^2 + \sum_{j=1}^{N} \frac{(b_j + c_j r)c_j r}{a_j}\hat{\varphi} - \sum_{j=1}^{N} \frac{b_j c_j^2 r^2}{a_j} = 0. \tag{A.30}$$

	S invader	E invader
$\lambda < \hat{\varphi}_{\text{res}}$	die out	die out
$\lambda = \hat{\varphi}_{\text{res}}$	die out	die out
$\lambda > \hat{\varphi}_{\text{res}}$	survive	survive

	S resident	E resident
$\lambda < \hat{\varphi}_{\text{new}}$	die out	die out
$\lambda = \hat{\varphi}_{\text{new}}$	die out	survive
$\lambda > \hat{\varphi}_{\text{new}}$	survive	cannot happen

Table A.1: Survival and extinction of sub-exponential (S) and exponential (E) invaders (appearing in the system at an infinitesimally small concentration) and residents (being present in a finite concentration) based on the relation between the intrinsic growth rate λ of the given species and the equilibrium value of the normalized production before the invasion ($\hat{\varphi}_{\text{res}}$) and after any change in the parameters or the composition of the system ($\hat{\varphi}_{\text{new}}$).

As all the parameters are positive, the $(+,+,-)$ sign pattern guarantees that there is a single positive root.

In the case of a single S-species ($N = 1$) and $m = 0$, denoting the parameters of this single species by a', b' and c', Eq. A.30 takes the form

$$\frac{c'r}{a'}\hat{\varphi}^2 + \frac{(b'+c'r)c'r}{a'}\hat{\varphi} - \frac{b'c'^2r^2}{a'} = 0, \tag{A.31}$$

where the positive root is the intrinsic growth rate of the given species:

$$\hat{\varphi} = \frac{-(b'+c'r) + \sqrt{b'^2 + c'^2r^2 + 6b'c'r}}{2} = \lambda'. \tag{A.32}$$

By introducing the notations $\vartheta_1 = \frac{c'r}{a'}$, $\vartheta_2 = \frac{(b'+c'r)c'r}{a'}$ and $\vartheta_3 = \frac{b'c'^2r^2}{a'}$, and using the previously introduced notations $\Theta_1 = \sum_{j=1}^{N} \frac{c_j r}{a_j}$, $\Theta_2 = \sum_{j=1}^{N} \frac{(b_j + c_j r)c_j r}{a_j}$ and $\Theta_3 = \sum_{j=1}^{N} \frac{b_j c_j^2 r^2}{a_j}$, Eqs. A.30 and A.31 take the following form:

$$(2m + \Theta_1)\hat{\varphi}^2 + \Theta_2\hat{\varphi} - \Theta_3 = 0 \tag{A.33}$$

$$\vartheta_1\hat{\varphi}^2 + \vartheta_2\hat{\varphi} - \vartheta_3 = 0. \tag{A.34}$$

The positive roots of the two equations are $\hat{\varphi}_{\text{res}}$ (the normalized production of the resident system) and λ' (the intrinsic growth rate of the invader), respectively. The sum of the two equations is

$$(2m + \Theta_1 + \vartheta_1)\hat{\varphi}^2 + (\Theta_2 + \vartheta_2)\hat{\varphi} - (\Theta_3 + \vartheta_3) = 0, \tag{A.35}$$

the positive root of which is the $\hat{\varphi}_{\text{new}}$ normalized production of the resulting system if the invasion was successful. It is easy to see that this root lies between the roots of Eqs. A.33 and A.34: since the only positive root of Eq. A.34 (corresponding to λ') is larger than the only positive root of Eq. A.33 (corresponding to $\hat{\varphi}_{\text{res}}$) in the case of successful invasion (see Eq. A.29), the parabola described by Eq. A.33 takes positive values and the parabola described by Eq. A.34 takes negative values in the range $(\hat{\varphi}_{\text{res}}, \lambda')$, while in $[0, \hat{\varphi}_{\text{res}})$ both parabolas are negative and above λ' both are positive, meaning that the positive root of the sum of the two parabolas described by Eq. A.35 must fall in the range $(\hat{\varphi}_{\text{res}}, \lambda')$. Thus,

$$\hat{\varphi}_{\text{res}} < \hat{\varphi}_{\text{new}} < \lambda', \tag{A.36}$$

showing that the equilibrium value of the normalized production of a purely sub-exponential system increases as a result of a successful invasion of an S-species. This is entirely in line with the expectation since the assumption of $\hat{\varphi}_{\text{new}} < \hat{\varphi}_{\text{res}}$ leads to contradiction: the decrease in the outflow due to the invasion would yield an increase in the concentration of all the resident species simultaneously with the establishment of the new species, but the overall concentration growth would mean a higher total production than the one before the invasion. Note that an increase in λ' (due to an increase in b' and/or c') shifts the positive root of the sum of the two parabolas to a larger value, i.e. increases $\hat{\varphi}_{\text{new}}$.

Although the success of the invasion (i.e., the invasiveness of the invader species) depends only on b' and c' and not on a' (since λ' calculated according to Eq. 5 in the main text does not depend on a'), the normalized production in the new equilibrium forming after a successful invasion depends on a' too, so the association rate of the invader scales its competitiveness: a lower association rate (a') results in higher normalized production ($\hat{\varphi}_{\text{new}}$), i.e. a higher chance of excluding species from the original system. Since ϑ_1, ϑ_2, $\vartheta_3 \sim 1/a'$, dividing a' with some positive constant results in a simple multiplication of the whole parabola described by Eq. A.34 with the same constant. Thus, when using a constant larger than 1 (i.e., when decreasing the association rate of the new species), all the negative parts of the parabola go downwards (while the roots do not change), which shifts the positive root of the summed parabola given by Eq. A.35 towards a larger value, i.e. closer to λ' and farther from $\hat{\varphi}_{\text{res}}$. Accordingly, for given $\hat{\varphi}_{\text{res}}$ and λ', the increase in a' increases the $\hat{\varphi}_{\text{new}} - \hat{\varphi}_{\text{res}}$ distance, enabling the exclusion of resident species of a larger range of intrinsic growth rate. As $a' \to \infty$, $\hat{\varphi}_{\text{new}} \to \hat{\varphi}_{\text{res}}$, while as $a' \to 0$, $\hat{\varphi}_{\text{new}} \to \lambda'$.

When a purely sub-exponential system is invaded by an exponential species ($a' = 0$), then $\hat{\varphi}_{\text{new}} = \lambda'$ (see Eq. A.22). According to Eq. A.29, such an invasion is successful if $\hat{\varphi}_{\text{res}} < \lambda'$, meaning that in this case $\hat{\varphi}_{\text{res}} < \hat{\varphi}_{\text{new}} = \lambda'$. If the resident system contains an E-species, then an exponential invader can spread only if its intrinsic growth rate is larger than that of the resident E-species, yielding again $\hat{\varphi}_{\text{res}} < \hat{\varphi}_{\text{new}} = \lambda'$. And finally, if a sub-exponential invader fulfills the criterion of Eq. A.29 and thereby manages to invade in a system where the normalized production is set by an E-species, then $\hat{\varphi}_{\text{new}} = \hat{\varphi}_{\text{res}}$ if the E-species does not go extinct with the appearance of the new species and $\hat{\varphi}_{\text{res}} < \hat{\varphi}_{\text{new}}$ otherwise (i.e., when the normalized production maintained by the coexisting sub-exponential species without the exponential one increases above the intrinsic growth rate of the exponential replicator).

A.6 The largest coexisting subset of a library of replicator species

As we have seen in Section A.4 and the main text, invasion will be successful if the intrinsic growth rate of the invader is greater than the normalized production of the resident community. This result can be used to determine which members of a given set of replicator species (characterized by parameter triplets $\{a_i, b_i, c_i\}$) will coexist in a system with a target replicator concentration m and a resource concentration r.

Without loss of generality, we assume that species 1 has the highest intrinsic growth rate, species 2 has the second highest, and so on, i.e. $\lambda_1 > \lambda_2 > \cdots > \lambda_N$. (Note that species having the same intrinsic growth rate are equivalent in terms of invasiveness, meaning that if one of them can spread in the system, then all of them can spread simultaneously.) It is intuitive that the replicator species with the highest intrinsic growth rate λ_1 can spread in the system the easiest. According to Eq. 8 in the main text or Eq. A.29, the species with the second highest intrinsic growth rate λ_2 can spread in the presence of the first species if $\lambda_2 > \hat{\varphi}(\{a_1, b_1, c_1\}; r, m)$. By repeating this procedure, the

largest set of coexisting species can be found by arranging the replicator species in the decreasing order of their intrinsic growth rates, and adding the subsequent species to the coexisting set one at a time, as long as their intrinsic growth rate meets the invasion criterion against the current normalized production. Since successful invasions cannot decrease the equilibrium value of the normalized production (Section A.5), thus the largest set of coexisting replicator species is the one that maximizes the value of $\hat{\varphi}$. Note that if an E-species is included in the coexisting set, then $\hat{\varphi}$ is equal to the intrinsic growth rate of that species, which is the smallest among the coexisting species.

A.6.1 Evolutionary stability: a serial invasion of species

In the absence of an analytical approach, here we analyze by numerical simulation the situation where different mutants enter the system sequentially. We have used here a 100-member random subset of the parameter space defined in the Methods section of the main text. Note that since the intrinsic growth rate of a given species does not depend on its association rate, different species may have the same growth rate even though the $\{a_i, b_i, c_i\}$ parameter triplets are different for all the species of the examined subset. Initially, the community contains only one species, namely the one with the lowest intrinsic growth rate (see Eq. 5 in the main text or Eq. A.11) from the subset of 100 species. At every 1000th time step, a random species from the subset is added to the community with an initial concentration of $m/10^5$. During the simulation, we measured the number of coexisting species (n), the normalized production of the system (φ), and the difference between the sum of the intrinsic growth rates of the actual set of species and the sum of the n largest intrinsic growth rates from the whole subset of 100 potential invaders. Figure A.1 shows these measures as the function of time.

The number of coexisting species increases and saturates at the value of 31 and in line with our previous results, the normalized production is a monotonically increasing function of the number of coexisting species. The final set of coexisting species is made up of the species with the 31 highest intrinsic growth rates. Consequently, the system selects for the highest intrinsic growth rates that are determined by the dissociation and replication rates at a given resource concentration r. The association rates have no effect on the composition of the final coalition as these values only affect the competitiveness, i.e. the ability to exclude a species being present during invasion, but the final coalition can no longer be invaded.

A.7 Ecological stability of coexisting replicators in the mass-regulated model

This section analyzes how the change in kinetic constants (association rate a_i, dissociation rate b_i and replication rate c_i) and the external parameters (target replicator concentration m, resource concentration r) affects the equilibrium characteristics (concentration of different species, normalized production) of a set of coexisting species. To simplify our analysis, we only allow one parameter to change at a time. We assume that the 'genotype' of the replicators is unaltered and the changes in the kinetic rates are due to the changes in external factors such as pH, temperature, etc. The response of the system to these changes characterizes the ecological stability. Only small perturbations are considered, where the number of coexisting species does not change, i.e. there is no extinction from the system. Section A.7.3 specifies the limits of the parameter changes set by the assumption of no extinction.

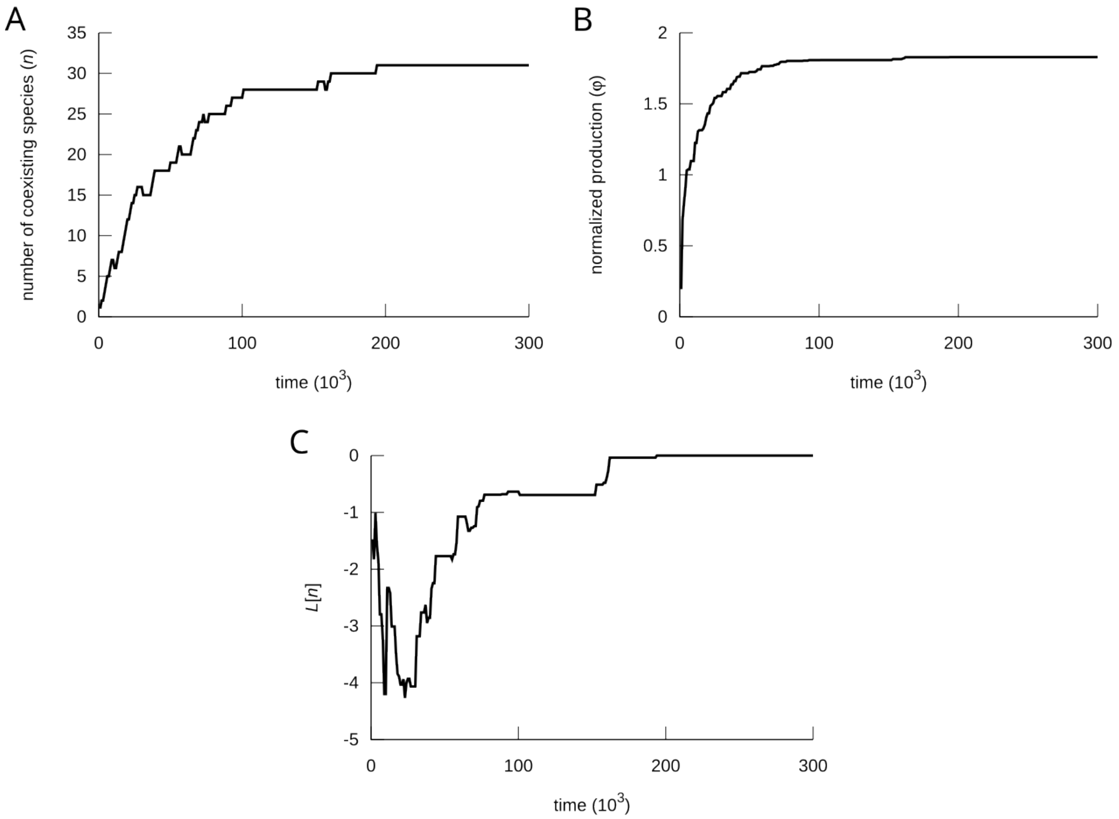

Figure A.1: Time evolution of the relevant measures during the serial invasion of species: number of coexisting species n (panel A), normalized production φ (panel B), difference between the sum of the growth rates of the actual set of coexisting species ($\sum_i \lambda_i$, where i runs over the species that are currently present), and the sum of the n largest intrinsic growth rates from the examined whole subset of species (denoted by $\tilde{\lambda}[n]$), i.e. $L[n] = \tilde{\lambda}[n] - \sum_i \lambda_i$ (panel C). The subset of species consists of 100 random species from the parameter space of the Methods section of the main text. The extinction limit, below which a species is considered extinct has been set to 10^{-5}. The invasion occurs at every 1000 time steps.

The top rows in the left panel of Table A.2 summarize the effects of increasing kinetic rates on the equilibrium of the system consisting only of S-species. As it can be seen, increasing the association rate a_i of a given species decreases its equilibrium concentration while increasing the equilibrium concentration of all other species (since the total replicator concentration is fixed). The effect of increasing the dissociation rate b_i or the replication rate c_i is the opposite: the growth of these rates increases the equilibrium concentration of the given species and decreases the equilibrium concentration of all the other species. Besides, increasing the association rate decreases the normalized production, whereas increasing the dissociation or replication rate increases it as a

simple consequence of the change in the ratio of double- vs. single-stranded replicators. For the proof, see Section A.7.1.

If the community contains an E-species (denoted by index 0) in addition to the S-species, the effect of changing the kinetic rates on the concentration is slightly different, see the top rows in the right panel of Table A.2. Increasing the association rate a_i of an S-species leads to a decrease in its own equilibrium concentration and an increase in the equilibrium concentration of the E-species, while the equilibrium value of the concentration of the other S-species and the normalized production remains unchanged. Increasing the dissociation rate b_i or the replication rate c_i of an S-species will increase its own equilibrium concentration, decrease the equilibrium concentration of the E-species and have no effect on the equilibrium value of the concentration of all other species and the normalized production (since the latter is defined by the E-species alone). Furthermore, an increase in the dissociation rate or the replication rate of the E-species (b_0 and c_0, respectively) causes an increase in the equilibrium concentration of this species, a decrease in the equilibrium concentration of all S-species, and an increase in the equilibrium value of the normalized production. The proof can be found in Section A.7.2.

Note that although altering the values of b and c produce the same qualitative changes in the system, their quantitative effect depends on the resource concentration: as the parameters c and r only appear together, as a product (cr) in the formulae, the system will be more sensitive to a change in b at low resource concentrations, and to a change in c at high resource concentrations.

The bottom rows of Table A.2 summarize the effect of small changes in the external parameters m and r on the equilibrium of a system consisting of only S-species (left panel) and sub-exponential species with an exponential one (right panel). The equilibrium concentration of a given S-species can be calculated according to Eq. A.14 or Eq. A.24 as

$$\hat{\mu}_i = \hat{x}_i + 2\hat{y}_i = \frac{c_i r}{a_i} \cdot \frac{-\hat{\varphi}^2 - (b_i + c_i r)\hat{\varphi} + b_i c_i r}{2\hat{\varphi}^2} = \frac{c_i^2 r^2 (b_i - \hat{\varphi}) - c_i r (b_i + \hat{\varphi})\hat{\varphi}}{2 a_i \hat{\varphi}^2}, \tag{A.37}$$

and the equilibrium concentration of an E-species can be expressed as $\hat{\mu}_0 = m - \sum_{i=1}^{N} \hat{\mu}_i$. The equilibrium value of the normalized production is a decreasing function of m in purely sub-exponential systems (see Eq. A.20), and is independent of m if an E-species is present (see Eq. A.22). Thus, using Eq. A.37, we can conclude that in a purely sub-exponential system, the equilibrium concentration of all species increases as the target replicator concentration m increases. If an E-species is also present, any increase in the target replicator concentration will be absorbed by the $\hat{\mu}_0$ concentration of the E-species, while the concentration of all the S-species remains unchanged. The effects of changes in resource concentration are less general. While an increase in r increases the equilibrium production of the system (see Eqs. A.20 and A.22), the change in the concentration of the different species is unambiguous, depending on the actual set of rate constants.

A.7.1 The impact of changing the parameters of a single species on the equilibrium of a purely sub-exponential system

As it is written in Eq. A.17, at the equilibrium of a system of S-species the normalized production $\hat{\varphi}$ satisfies the following equation:

$$\left[2m + \sum_{j=1}^{N} \frac{c_j r}{a_j}\right] \hat{\varphi}^2 + \sum_{j=1}^{N} \frac{(b_j + c_j r) c_j r}{a_j} \hat{\varphi} - \sum_{j=1}^{N} \frac{b_j c_j^2 r^2}{a_j} = 0. \tag{A.38}$$

	$\hat{\mu}_i$	$\hat{\mu}_j$	$\hat{\varphi}$
$a_i\uparrow$	$\downarrow$	$\uparrow$	$\downarrow$
$b_i, c_i\uparrow$	$\uparrow$	$\downarrow$	$\uparrow$
$m\uparrow$	$\uparrow$	$\uparrow$	$\downarrow$
$r\uparrow$	$\updownarrow$	$\updownarrow$	$\uparrow$

	$\hat{\mu}_i$	$\hat{\mu}_j$	$\hat{\mu}_0$	$\hat{\varphi}$
$a_i\uparrow$	$\downarrow$	—	$\uparrow$	—
$b_i, c_i\uparrow$	$\uparrow$	—	$\downarrow$	—
$b_0, c_0\uparrow$	$\downarrow$	$\downarrow$	$\uparrow$	$\uparrow$
$m\uparrow$	—	—	$\uparrow$	—
$r\uparrow$	$\updownarrow$	$\updownarrow$	$\updownarrow$	$\uparrow$

Table A.2: The direction of change in the equilibrium values of the replicator concentrations ($\hat{\mu}_i$, $\hat{\mu}_j$, $\hat{\mu}_0$) and the normalized production ($\hat{\varphi}$) as a result of increasing the parameters a, b, and c (top rows), the target replicator concentration m, or the resource concentration r (bottom rows). This is shown both for purely sub-exponential systems (left table) and systems with an E-species, indexed by 0 (right table). Only a single parameter is altered at a time. When changing a parameter of an S-species i, the index j refers to any of the other, unperturbed S-species. Notations: $\uparrow$ and $\downarrow$ denote increase and decrease, respectively; $\updownarrow$ means ambiguous change (increase or decrease, depending on the parameters of the actual community).

Using the notations

$$\vartheta_{1,i} = \frac{c_i r}{a_i}, \quad \vartheta_{2,i} = \frac{(b_i + c_i r)c_i r}{a_i}, \quad \vartheta_{3,i} = \frac{b_i c_i^2 r^2}{a_i}, \tag{A.39}$$

Eq. A.38 can be rewritten as

$$\vartheta_{1,i}\hat{\varphi}^2 + \vartheta_{2,i}\hat{\varphi} - \vartheta_{3,i} + \left[2m + \sum_{j=1,j\neq i}^{N} \vartheta_{1,j}\right]\hat{\varphi}^2 + \sum_{j=1,j\neq i}^{N} \vartheta_{2,j}\hat{\varphi} - \sum_{j=1,j\neq i}^{N} \vartheta_{3,j} = 0, \tag{A.40}$$

which can be considered as a sum of two parabolas given by the equations $\vartheta_{1,i}\hat{\varphi}^2 + \vartheta_{2,i}\hat{\varphi} - \vartheta_{3,i} = 0$ and $\left[2m + \sum_{j=1,j\neq i}^{N} \vartheta_{1,j}\right]\hat{\varphi}^2 + \sum_{j=1,j\neq i}^{N} \vartheta_{2,j}\hat{\varphi} - \sum_{j=1,j\neq i}^{N} \vartheta_{3,j} = 0$. The value of $\hat{\varphi}$ is given by the positive root of the summed parabola, while the positive root of the first parabola corresponds to λ_i and the positive root of the second parabola (being smaller than λ_i, since otherwise species i could not be present, see Eq. A.36) corresponds to the equilibrium value of the normalized production in the absence of species i replicators. According to Section A.5, as the association rate a_i decreases (thereby stretching the first parabola), the root of the summed parabola increases. So we can conclude that an increase in a_i leads to a decrease in $\hat{\varphi}$.

Changing the dissociation rate b_i or the replication rate c_i changes the positive root of the first parabola $\vartheta_{1,i}\hat{\varphi}^2 + \vartheta_{2,i}\hat{\varphi} - \vartheta_{3,i} = 0$ given by the intrinsic growth rate $\lambda_i = \frac{-(b_i+c_i r)+\sqrt{b_i^2+c_i^2r^2+6b_ic_ir}}{2}$ (cf. Eq. A.32), while the positive root of the second parabola $\left[2m + \sum_{j=1,j\neq i}^{N} \vartheta_{1,j}\right]\hat{\varphi}^2 + {}+\sum_{j=1,j\neq i}^{N} \vartheta_{2,j}\hat{\varphi} - \sum_{j=1,j\neq i}^{N} \vartheta_{3,j} = 0$ remains the same (somewhere below λ_i). An increase in b_i or c_i makes λ_i larger, which, by extending the range at which the first parabola takes negative values while leaving the second parabola unaltered, extends the range at which the summed parabola takes negative values, i.e. shifts the positive root of the summed parabola to a larger value. Consequently, an increase in b_i or c_i leads to an increase in $\hat{\varphi}$.

But what happens with the species concentrations due to a change in $\hat{\varphi}$? With the rearrangement

of Eq. A.37, it can be shown that besides Eq. A.38, the equation

$$\left[2\hat{\mu}_i + \frac{c_i r}{a_i}\right]\hat{\varphi}^2 + \frac{c_i r}{a_i}(b_i + c_i r)\hat{\varphi} - \frac{b_i c_i^2 r^2}{a_i} = 0 \tag{A.41}$$

is also true for $\hat{\varphi}$ at any species index i. With the notations given by Eq. A.39, Eq. A.41 can be written in a form similar to Eq. A.18, namely as

$$\left[2\hat{\mu}_i + \vartheta_{1,i}\right]\hat{\varphi}^2 + \vartheta_{2,i}\hat{\varphi} - \vartheta_{3,i} = 0. \tag{A.42}$$

We know that $\hat{\varphi}$ is a solution of Eq. A.42 for any species index i even after changing a parameter of species i. For any $j \neq i$, the change in a_i, b_i or c_i does not change $\vartheta_{1,j}$, $\vartheta_{2,j}$ and $\vartheta_{3,j}$, meaning that for $j \neq i$, only $\hat{\varphi}$ and $\hat{\mu}_j$ are affected in Eq. A.42 by the change of a parameter of species i. To satisfy Eq. A.42 at a smaller $\hat{\varphi}$ value and the same $\vartheta_{1,j}$, $\vartheta_{2,j}$, $\vartheta_{3,j}$ values, $\hat{\mu}_j$ has to be larger, meaning that a decrease in $\hat{\varphi}$ caused by the change of a_i, b_i or c_i increases $\hat{\mu}_j$ for any $j \neq i$. And since $m = \sum_{j=1}^{N} \hat{\mu}_j$ is always true, the increase in the total concentration of all the other species implies a decrease in $\hat{\mu}_i$.

To sum up, as it is shown by the left panel of Table A.2, in the absence of any E-species, an increase in a_i leads to a decrease in $\hat{\varphi}$ and $\hat{\mu}_i$ and an increase in $\hat{\mu}_j$ for any $j \neq i$, while an increase in b_i or c_i yields an increase in $\hat{\varphi}$ and $\hat{\mu}_i$ and a decrease in $\hat{\mu}_j$ for any $j \neq i$.

A.7.2 The impact of changing the parameters of a single species on the equilibrium of a system containing an E-species

In the presence of an E-species (having an association rate $a_0 = 0$), the normalized production at the equilibrium is determined by the intrinsic growth rate λ_0 of the E-species (see Eq. A.22), and therefore, changing the parameters of any S-species i has no impact on $\hat{\varphi}$. Accordingly, using that the total equilibrium concentration of any S-species is given by Eq. A.37, it is easy to see that changing a_i, b_i or c_i affects only $\hat{\mu}_i$ and does not influence $\hat{\mu}_j$ for any $j \neq i$. Nevertheless, the equilibrium concentration of the E-species does not remain unaltered when a parameter of an S-species changes: to keep the total replicator concentration of the system at the fixed equilibrium value of m, an increase in $\hat{\mu}_i$ is compensated by a decrease in $\hat{\mu}_0$ and a decrease in $\hat{\mu}_i$ yields an increase in $\hat{\mu}_0$.

The dependence of $\hat{\mu}_i$ on a_i is obvious from Eq. A.37: larger values of a_i yield smaller values of $\hat{\mu}_i$. The impact of changing b_i or c_i on $\hat{\mu}_i$ can be seen from Eq. A.42 after the following rearrangement:

$$2\hat{\mu}_i\hat{\varphi}^2 + \vartheta_{1,i}\hat{\varphi}^2 + \vartheta_{2,i}\hat{\varphi} - \vartheta_{3,i} = 0. \tag{A.43}$$

This equation can be interpreted as the sum of two parabolas that are given by the formulas $2\hat{\mu}_i\hat{\varphi}^2 = 0$ and $\vartheta_{1,i}\hat{\varphi}^2 + \vartheta_{2,i}\hat{\varphi} - \vartheta_{3,i} = 0$. The positive root of the latter parabola is λ_i (see Eq. A.34), which is an increasing function of b_i and c_i. The positive root of the summed parabola given by Eq. A.43 is the whole system's $\hat{\varphi}$ value, which is set by the E-species to λ_0. Thus, to keep the root of the summed parabola unaltered, in the case of increasing b_i or c_i and thereby λ_i, the first parabola $2\hat{\mu}_i\hat{\varphi}^2 = 0$ must be stretched, which can be realized by an increase in $\hat{\mu}_i$.

But what happens if we change the dissociation or the replication rate of the E-species instead of an S-species? The growth of both b_0 and c_0 increases λ_0 (see Eq. A.22). Using again Eq. A.43, one can see that for an unaltered λ_i root of the parabola $\vartheta_{1,i}\hat{\varphi}^2 + \vartheta_{2,i}\hat{\varphi} - \vartheta_{3,i} = 0$ and an increased λ_0 root of the parabola $\left[2\hat{\mu}_i + \vartheta_{1,i}\right]\hat{\varphi}^2 + \vartheta_{2,i}\hat{\varphi} - \vartheta_{3,i} = 0$, $\hat{\mu}_i$ must be decreased, meaning that the

increase in λ_0 and thereby in the equilibrium normalized production of the system must imply a decrease in $\hat{\mu}_i$ for any $i \neq 0$. In the meantime, $\hat{\mu}_0 = m - \sum_{i=1}^{N} \hat{\mu}_i$ increases together with the equilibrium normalized production.

To sum up, as it is shown by the right panel of Table A.2, in the presence of an E-species indexed by 0, an increase in the association rate a_i of any S-species of $0 < i$ yields a decrease in $\hat{\mu}_i$, an increase in $\hat{\mu}_0$ and no change in $\hat{\varphi}$ and in $\hat{\mu}_j$ for any $0 < j \neq i$, whereas an increase in b_i or c_i of any S-species of $0 < i$ leads to an increase in $\hat{\mu}_i$, a decrease in $\hat{\mu}_0$ and no change in $\hat{\varphi}$ and in $\hat{\mu}_j$ for any $0 < j \neq i$. Besides, an increase in the parameters b_0 or c_0 of the E-species yields an increase in $\hat{\varphi}$ and $\hat{\mu}_0$ and a decrease in $\hat{\mu}_i$ for any $0 < i$.

A.7.3 The critical parameter values that lead to the first extinction in a given coalition

Along Section A.7 and in Table A.2, we have assumed that the perturbation of the parameters is small enough not to change the set of coexisting species. As the strength of the perturbation increases, there will be a 'critical' value for the external parameters (m^*, r^*) and for each rate constant (a_i^*, b_i^*, c_i^*) at which the first extinction (not necessarily of species i) occurs. In most of the cases, there is no simple analytical form for these critical values, but they can be calculated by an exhaustive search.

In a purely sub-exponential system, the equilibrium concentration $\hat{\mu}_i$ is given by Eq. A.37 for any species i. Thus, e.g. the critical association rate of a given species i can be obtained by calculating for each present species j the association rate a_i corresponding to zero equilibrium concentration $\hat{\mu}_j$. Among the obtained values, the one that is the closest to the original association rate of species i is the 'critical' association rate a_i^* of species i at which the first extinction occurs. Another option is to find a_i^* as that setting of a_i at which the equilibrium normalized production $\hat{\varphi}$ of the system (given by Eq. A.20) first becomes equal to the intrinsic growth rate $\lambda_j = \frac{-(b_j+c_jr)+\sqrt{b_j^2+c_j^2r^2+6b_jc_jr}}{2}$ (Eq. 5 in the main text) of any present species j. The b_i^* and c_i^* values can be determined the same way.

In the presence of an E-species indexed by 0, the equilibrium normalized production $\hat{\varphi}$ is fixed by the E-species to its intrinsic growth rate λ_0 (see Eq. A.22), which is the lowest intrinsic growth rate among the coexisting species. In this case, the equilibrium concentration $\hat{\mu}_i$ of an S-species i (given by Eq. A.37) can not be set to 0 by tuning its association rate $a_i > 0$. Here $a_i^* = 0$ for any $i \neq 0$, i.e. the first extinction due to the change in a_i occurs when species i becomes exponential: at this point, since $\lambda_0 < \lambda_i$ for any $i \neq 0$, the original E-species denoted by 0 will be excluded.

The adjustment of the dissociation rate b_i or the replication rate c_i of any S-species i can lead to the extinction of either the given S-species itself or the E-species of the coalition. When decreasing b_i or c_i, λ_i decreases and species i dies out ($\hat{\mu}_i$ in Eq. A.37 becomes 0) at $\lambda_i = \hat{\varphi}$, i.e. at $\lambda_i = \lambda_0$. When increasing b_i or c_i, the E-species dies out ($\hat{x}_0 = 0$ and $\hat{y}_0 = 0$ in Eq. A.25) at that b_i^* or c_i^* value for which $\lambda_0 - \frac{r}{m}\sum_{i=1}^{N} c_i\hat{x}_i = 0$, i.e. when the equilibrium normalized production maintained by the S-species (indexed by $i = 1, 2 \ldots N$) in the absence of the E-species (which increases with the increment of λ_i, and can also be calculated according to Eq. A.20) reaches the intrinsic growth rate of the E-species. Similarly, when decreasing the dissociation rate or the replication rate of the E-species, the E-species dies out ($\hat{\mu}_0 = 0$) at that b_0^* or c_0^* value for which $\lambda_0 = \frac{r}{m}\sum_{i=1}^{N} c_i\hat{x}_i$, i.e. when the decreasing intrinsic growth rate of the E-species reaches the equilibrium normalized production maintained by the given set of S-species in the absence of the E-species. On the other hand, increasing b_0 or c_0 leads to the extinction of an S-species ($\hat{\mu}_i = 0$) when the increasing equilibrium normalized production $\hat{\varphi}$ set by the E-species reaches the intrinsic growth rate λ_i of

the given S-species, i.e. at $\lambda_0 = \lambda_i$.

For the critical target replicator concentration m^* at which the first extinction occurs as m decreases, there is a simple analytical formula in a purely sub-exponential system. Here m^* can be formalized from the condition $\hat{\varphi}(m^*) = \tilde{\lambda}$, where $\tilde{\lambda} = \min \lambda_j$. (The same formula for m^* can be obtained by searching for the largest target replicator concentration at which the equilibrium concentration $\hat{\mu}_i$ of any present species i goes to zero.) The result is (cf. Eq. A.18)

$$m^* = \frac{-\Theta_1 \tilde{\lambda}^2 - \Theta_2 \tilde{\lambda} + \Theta_3}{2\tilde{\lambda}^2}, \tag{A.44}$$

where the Θ values can be calculated according to Eq. A.19. m^* is also the critical total replicator concentration at which a species characterized by an intrinsic growth rate of $\tilde{\lambda}$ can enter the system as m increases. Therefore, it does not matter whether the constants of the species in question are included in the calculation of the Θ values (according to Eq. A.34, their contribution to m^* is zero). However, it is important to note that m^* refers to a given set of species, so the Θ parameters must be recalculated after each change in the composition of the coalition.

In the presence of an E-species, $\hat{\varphi}$ does not depend on m (see Eq. A.22) and thus, the equilibrium concentration of any S-species is also independent of m (see Eq. A.37). Therefore, a decrease in the target replicator concentration decreases the concentration of the E-species only, so it will die out first, determining m^*. The extinction of the E-species ($\hat{\mu}_0 = 0$) happens when m reduces to the sum of the equilibrium concentrations of all the S-species, which are given by Eq. A.37.

Regarding the other external parameter given by the resource concentration r, the critical value r^* at which the first extinction occurs (or at which a given species can enter the system) has got no simple analytical form. It can be calculated by an exhaustive search: either by tuning r until the equilibrium concentration of any species (either sub-exponential or exponential) first becomes 0, or by finding the resource concentration at which the equilibrium normalized production equals the intrinsic growth rate of any present species. The cause of the analytical intractability — in contrast to the case of the target replicator concentration m — is twofold: first, r cannot be expressed from the equilibrium normalized production formula, whereas m is only present in the prefactor of the quadratic term in Eq. A.18; and second, the intrinsic growth rate of the replicator species (which does not depend on m) is also a function of r.

A.8 Parabolic dynamics of a single species at high total concentration

Here we show that in the case of a single S-species at high total concentration, the dynamics are parabolic, i.e., the system's total production is proportional to the square root $\sqrt{\mu}$ of its total concentration. By Eq. A.20, the total equilibrium production of the system for a single replicator species, in the limit of large m, is

$$\lim_{m \to \infty} m\hat{\varphi} = m\sqrt{\frac{\Theta_3}{2m}} = cr\sqrt{\frac{b}{2a}}\sqrt{m}, \tag{A.45}$$

where we used $\Theta_3 = bc^2r^2/a$ (Eq. A.19). Then, since $m = \mu$ at equilibrium, the total concentration obeys

$$\frac{\mathrm{d}\mu}{\mathrm{d}t} \approx \left(rc\sqrt{\frac{b}{2a}} \right) \sqrt{\mu}. \tag{A.46}$$

This straightforwardly explains why production is approximately proportional to the square root of the mass in experimental systems in the large total mass limit [3, 4, 5]. The solution to the differential equation in Eq. A.46 is quadratic in time, which is why it is called parabolic growth.

A.9 Diversity-maintaining ability in the abundance of resources

In the high abundance of resources, when rc_i is very high for any replication rate c_i, the dissociation rate b_i decides which species grows the fastest and eventually one species excludes all others. In a purely sub-exponential system, this can be seen from Eqs. 5 and 8 in the main text and Eq. A.20: at $r \to \infty$, λ_i tends to b_i and $\hat{\varphi}$ tends to a weighted arithmetic mean of the dissociation rates (namely to $\frac{\sum_{j=1}^{N} \frac{b_j c_j^2}{a_j}}{\sum_{j=1}^{N} \frac{c_j^2}{a_j}}$, see Section A.3.1), which would fall between the b_i values of the N different species assumed to live together, contradicting the current criterion of survival ($b_i > \hat{\varphi}$) for at least one of the species of the supposed coalition, meaning that at $r \to \infty$, the only case not leading to contradiction is the presence of a single species (i.e., $N = 1$), namely the one with the largest dissociation rate $b_{\max}$. (Note that the intrinsic growth rate of the final survivor is always larger than the normalized production at $r \to \infty$, since $\lambda_{\max}$ is exactly the value that $\hat{\varphi}$ takes at $N = 1$ number of species and $m = 0$ (cf. Eq. A.32), and $\hat{\varphi}$ is a decreasing function of m.)

When increasing the resource concentration r in a system that contains both sub-exponential species (indexed by $j = 1, 2 \ldots N$) and an exponential one (indexed by 0), either the exponential species dies out at some point, leaving behind a purely sub-exponential coalition from which at $r \to \infty$ only the one having the highest dissociation rate $b_{\max}$ survives, or the exponential species excludes all the other species. According to Eq. A.25, the E-species goes extinct ($\hat{x}_0 = 0$, $\hat{y}_0 = 0$) when the normalized production of the given set of S-species $\frac{r}{m} \sum_{j=1}^{N} c_j \hat{x}_j$ increases to the single normalized production value at which the E-species can be at equilibrium, namely $\hat{\varphi} = \lambda_0$ (see Eq. A.22). When $\hat{\mu}_0$ reaches 0, the system becomes purely sub-exponential, for which the equilibrium normalized production is given by Eq. A.20. Since $\hat{\varphi}$ changes continuously, at the extinction of the E-species

$$\lambda_0 = \frac{-\Theta_2 + \sqrt{\Theta_2^2 + 4(2m + \Theta_1)\Theta_3}}{2(2m + \Theta_1)}, \tag{A.47}$$

where λ_0 and Θ_1, Θ_2, Θ_3 are given as a function of the kinetic rates and the r resource concentration by Eqs. A.22 and A.19, respectively.

In the limit of $r \to \infty$, Eq. A.47 can be written as

$$b_0 = \frac{\sum_{j=1}^{N} \frac{c_j^2}{a_j} b_j}{\sum_{j=1}^{N} \frac{c_j^2}{a_j}}, \tag{A.48}$$

where the right side is a simple weighted arithmetic mean of the dissociation rates of the S-species. Denoting it as $\bar{b}_{\text{sub}}$, we can conclude that $b_0 < \bar{b}_{\text{sub}}$ means that the normalized production at which the E-species is capable of maintaining a positive equilibrium concentration is exceeded by the normalized production of the given set of S-species already at a finite resource concentration, and thus, the E-species can not be present at $r \to \infty$. Thus, the case of $b_0 < \bar{b}_{\text{sub}}$ leads back to the purely sub-exponential case, enabling the survival of only the fastest dissociating species (characterized by $b_{\max}$) at $r \to \infty$.

On the other hand, if $\overline{b}_{\text{sub}} \leq b_0$ (which does not necessarily mean that the highest dissociation rate in the considered system is b_0), it means that the E-species is still present at $r \to \infty$ and the whole sub-exponential species set considered in $\overline{b}_{\text{sub}}$ can not coexist with it, as the condition $\hat{\varphi} < \lambda_j$ of the survival of an S-species j (see Table A.1), which can be written as $b_0 < b_j$ at $r \to \infty$ in the presence of an E-species, contradicts the presumption of $\overline{b}_{\text{sub}} \leq b_0$ for at least one of the considered S-species since $\min(b_j) \leq \overline{b}_{\text{sub}}$. Knowing that the whole sub-exponential species set considered in $\overline{b}_{\text{sub}}$ can not coexist with the E-species, one can reduce the set of examined S-species (removing the species of small dissociation rates) and re-calculate $\overline{b}_{\text{sub}}$. If the removal of S-species yields a sub-exponential species set for which $b_0 < \overline{b}_{\text{sub}}$ (i.e. if $b_{\max} \neq b_0$), it means that the E-species eventually dies out, and then the S-species having the highest dissociation rate excludes all others. Otherwise, i.e. if $b_{\max} = b_0$, the E-species remains in the system at $r \to \infty$, excluding all the S-species. All things considered, at $r \to \infty$ always only a single species is present, namely the one having the largest dissociation rate $b_{\max}$, and it does not matter whether it is sub-exponential or exponential.

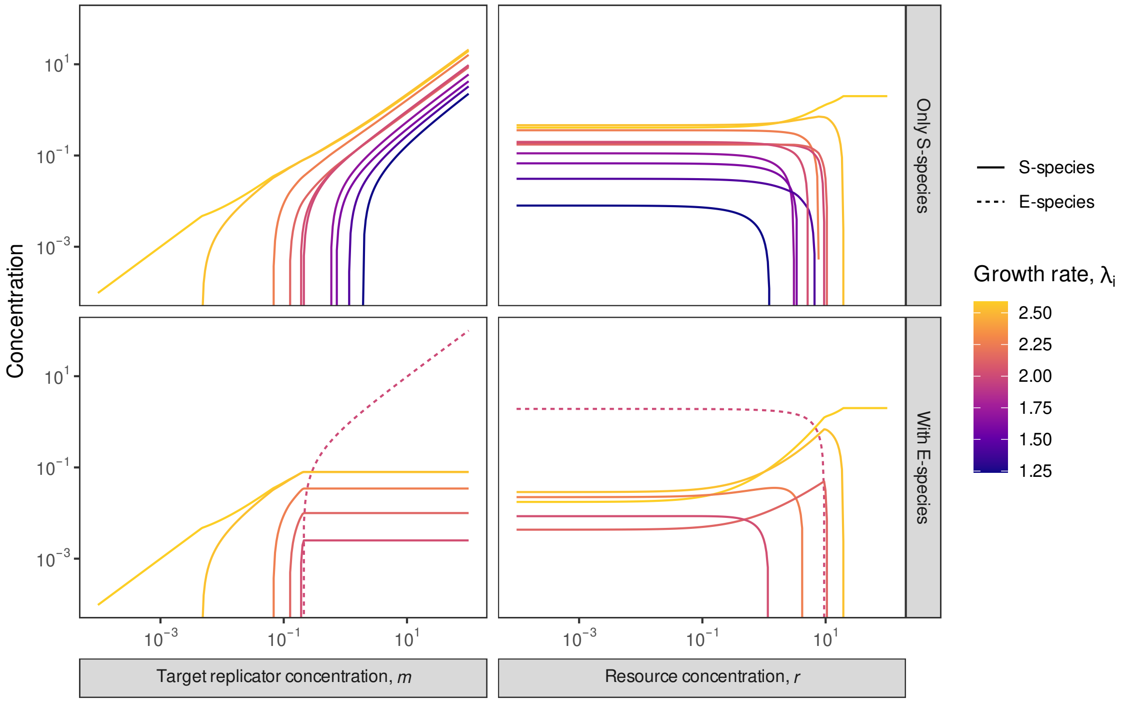

Figure A.2: As Fig. 2A in the main text, except with *absolute* instead of relative equilibrium species concentrations along the ordinate.

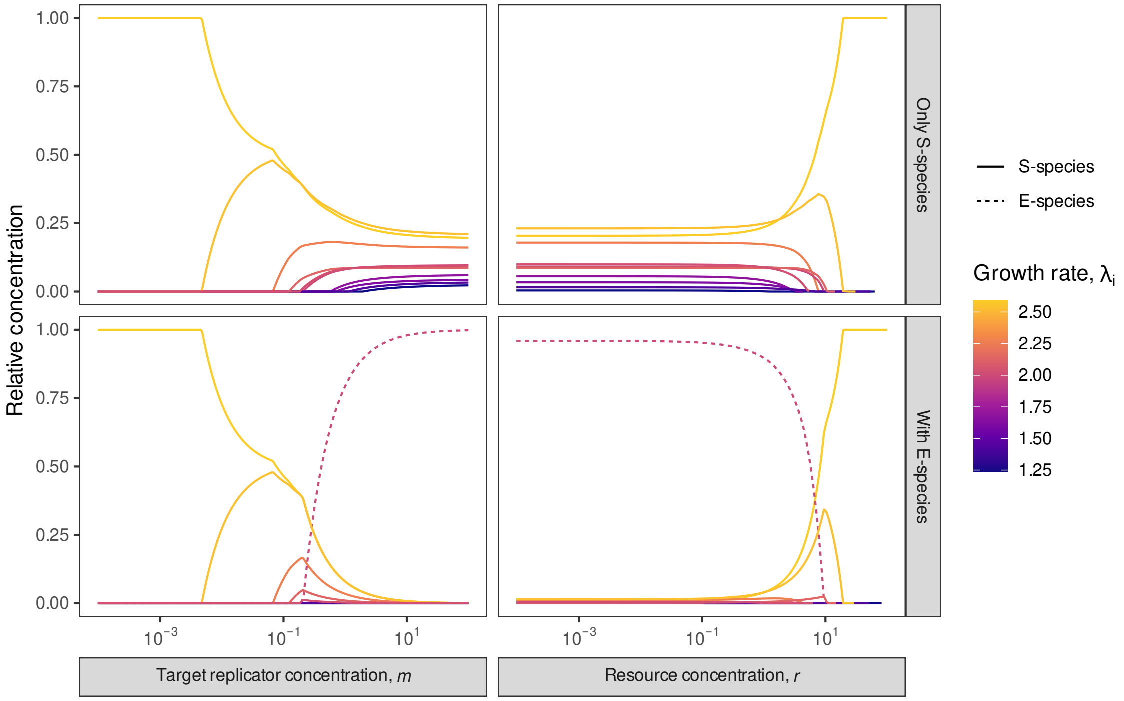

Figure A.3: As Fig. 2A in the main text, except with equilibrium relative species concentrations depicted on a *linear* instead of a logarithmic scale along the ordinate.

Appendix B: Resource-regulated model

Besides the mass-regulated model analyzed in the main text and in Appendix A, another option in a mechanistic model of template-directed replication is the assumption of a chemostat-type system, where the resource concentration is not fixed but has its own dynamics, and instead of applying a direct regulation on the system's total concentration, it is regulated by a constant inflow of a solution of the resource R and a constant outflow. Using as the external parameters of the system the dilution rate φ (defined as the volume of the outflow or, equivalently, the inflow in a time step divided by the constant volume of the system) and the resource concentration ρ in the inflow, the dynamics of the single- and the double-stranded replicator concentrations (x_i and y_i) of replicator species i and the time evolution of the r resource concentration of the system can be described as

$$\begin{aligned} \dot{x_i} &= -2a_i x_i^2 + 2b_i y_i - c_i r x_i - \varphi x_i \\ \dot{y_i} &= a_i x_i^2 - b_i y_i + c_i r x_i - \varphi y_i \\ \dot{r} &= -r \sum_{i=1}^{N} c_i x_i + (\rho - r)\,\varphi, \end{aligned} \tag{B.1}$$

where the dot denotes differentiation with respect to time (e.g. $\dot{x}_i = \mathrm{d}x_i/\mathrm{d}t$). The last term of the equations guarantees that the total concentration $r + \sum_{j=1}^{N} x_j + 2y_j$ is set to ρ at equilibrium (where $\dot{r} + \sum_{j=1}^{N} \dot{x}_j + 2\dot{y}_j = 0$). Therefore, in the following we refer to ρ as the equilibrium total concentration.

Since the production of single- and double-stranded replicators are described by the same terms in Eq. B.1 as in Eq. A.1, the dynamical matrix written in Eq. A.8 for the mass-regulated model can be used in the resource-regulated model too, when considering a given r value of the resource concentration. Thus, one can define the intrinsic growth rate the same way in the resource-regulated model as in the mass-regulated model (Eq. A.11), namely as

$$\lambda_i(r) = \frac{-(b_i + c_i r) + \sqrt{b_i^2 + c_i^2 r^2 + 6 b_i c_i r}}{2}, \tag{B.2}$$

noting that it is not a constant in the resource-regulated model but, being a function of the time-dependent resource concentration, a variable that changes in time. At a given r value, $\lambda_i(r)$ defines the growth rate (or per capita production) of species i at its infinitesimally small concentration or, if species i is exponential, then its growth rate when being present in an arbitrary amount.

The following subsections present the analysis of the resource-regulated model. The principle behind the equilibrium state (Section B.1) and the invasion criterion (Section B.2) is the same in the resource-regulated model as in the mass-regulated model, namely the equilibrium is reached when the growth rate of all species becomes equal to the relative outflow and the invasion is successful if the intrinsic growth rate of the invader is larger than the outflow rate of the resident system. Nevertheless, while in the mass-regulated model the outflow of the system changes together with the replicator concentrations and it is determined by the production of the replicators, the growth rate of each species adjusts in the resource-regulated model to the fixed relative outflow (dilution rate), which does not change even when the coalition composition is altered: the increase in $\hat{\varphi}$ due to successful invasions is replaced in the resource-regulated model by a decrease in the equilibrium resource concentration $\hat{r}$ (Section B.3). Furthermore, in the mass-regulated model the intrinsic growth rates are fixed by the constant resource concentration and the changes in the invasibility

of the system at given external parameters originate from changes in the outflow, whereas in the resource-regulated model, through the ever-changing resource concentration of the system, the λ_i values are those that depend on the current composition of the coalition and not the outflow. Hence, in the resource-regulated model instead of the intrinsic growth rate (that can be interpreted in the mass-regulated model as that 'critical' value of the equilibrium normalized production below which the given species can invade an established community), it is easier to work with the critical resource concentration (see Eq. B.22) corresponding to that value of the resource concentration of an established community above which the given species can invade. Just like the intrinsic growth rates in the mass-regulated model, the critical resource concentrations can be used in the resource-regulated model for selecting the coexisting ones from a given set of replicator species at given external parameters of the system (Section B.4).

Regarding the response of an established community to small perturbations of the model parameters (Section B.5), the behavior of the resource-regulated model is similar to that of the mass-regulated model, but due to the fixed outflow, a change in $\hat{\varphi}$ observed in the mass-regulated model is replaced in the resource-regulated model by a change in $\hat{r}$ in the opposite direction. An increase in the external parameter m in the mass-regulated model acts similarly to an increase in ρ in the resource-regulated model, whereas an increase in the other external parameter r of the mass-regulated model has similar effects as a decrease in φ in the resource-regulated model. From the viewpoint of the wide-range dependence of the coalition composition on the external model parameters (Section B.6), the resource-regulated model is again very similar in nature to the mass-regulated model: a decrease in ρ moderates the diversity-maintaining ability of the chemostat (with the gradual extinction of the species having larger critical resource concentrations), and an increase in φ also leads to a gradual loss of species (but here, due to the φ-dependence of the critical resource concentrations, the species being the most endangered can be different at different values of φ — just like how the order of intrinsic growth rates can vary throughout the r-screen in the mass-regulated model).

B.1 Coexistence of different replicator species

In this section, we present the single- and double-stranded replicator concentrations at the equilibrium of the system of Eq. B.1 for pure sub-exponential systems and for a single exponential species coexisting with sub-exponential species. (If there is more than one E-species, the fittest one will exclude all others.) The equilibrium is the state where the concentrations do not change because the growth rates are equal to the fixed per unit outflow of the system for each species, i.e. where

$$\varphi = \frac{\hat{r} c_i \hat{x}_i}{\hat{\mu}_i} \tag{B.3}$$

for all i (cf. Eq. A.5). Besides, since at equilibrium the resource concentration cannot change either, it is also true that

$$\varphi = \frac{\rho\varphi - \hat{r}\sum_{i=1}^{N} c_i \hat{x}_i}{\hat{r}}, \tag{B.4}$$

expressing that the incoming resource not utilized by the replicators flows out from the equilibrium system.

B.1.1 Coexistence of S-species

Treating the equilibrium resource concentration $\hat{r}$ as a known constant, we get N independent equation pairs from Eq. B.1, from which the steady state value of any S-species i can be expressed as

$$\begin{aligned}\hat{x}_i &= \frac{-\varphi^2 - (b_i + c_i\hat{r})\varphi + b_i c_i \hat{r}}{2a_i\varphi} = \frac{c_i\hat{r}(b_i - \varphi) - \varphi(b_i + \varphi)}{2a_i\varphi} \\ \hat{y}_i &= \frac{\varphi^3 + b_i\varphi^2 - c_i\hat{r}(c_i\hat{r} + 2b_i)\varphi + b_i c_i^2 \hat{r}^2}{4a_i\varphi^2} = \frac{[c_i\hat{r}(b_i - \varphi) - \varphi(b_i + \varphi)]\,(c_i\hat{r} - \varphi)}{4a_i\varphi^2},\end{aligned} \tag{B.5}$$

which is the same as Eq. A.14 with φ and $\hat{r}$ substituting $\hat{\varphi}$ and r, respectively.

Thus, the ratio of double- vs. single-stranded replicators of an S-species indexed by i can be expressed in terms the of relative outflow as

$$\frac{\hat{y}_i}{\hat{x}_i} = \frac{c_i\hat{r} - \varphi}{2\varphi}, \tag{B.6}$$

similarly to Eq. A.15, but using the input parameter φ instead of the $\hat{\varphi}$ value set by the system itself.

The equilibrium concentration $\hat{r}$ of the resource can be obtained from $\dot{r} = 0$, substituting in $\dot{r}$ from Eq. B.1 and $\hat{x}_i$ from Eq. B.5, yielding

$$\sum_{i=1}^{N} \frac{c_i^2(b_i - \varphi)}{2a_i\varphi}\hat{r}^2 + \left[\varphi - \sum_{i=1}^{N}\frac{c_i(b_i + \varphi)}{2a_i}\right]\hat{r} - \varphi\rho = 0. \tag{B.7}$$

Using the notations

$$\Theta_1 = \sum_{i=1}^{N} \frac{c_i^2(b_i - \varphi)}{2a_i\varphi}, \quad \Theta_2 = \sum_{i=1}^{N}\frac{c_i(b_i + \varphi)}{2a_i}, \tag{B.8}$$

Eq. B.7 can be rewritten as

$$\Theta_1\hat{r}^2 + [\varphi - \Theta_2]\,\hat{r} - \varphi\rho = 0. \tag{B.9}$$

The positive root of this equation is

$$\hat{r} = \frac{\Theta_2 - \varphi + \sqrt{\varphi^2 - 2\varphi\Theta_2 + \Theta_2^2 + 4\Theta_1\varphi\rho}}{2\Theta_1}. \tag{B.10}$$

This can be substituted in the formulas of Eq. B.5 to express the equilibrium concentrations purely as a function of the model parameters. According to Eq. B.10, the equilibrium resource concentration $\hat{r}$ is jointly determined by all the existing replicators species, and it is an increasing function of the equilibrium total concentration ρ and the dilution rate φ. At $\rho \to 0$, $\hat{r}$ asymptotically goes to 0, making the survival of any species impossible. Too large dilution rates (i.e. when the outflow is faster than the dissociation or the replication of the species, see Section B.2) also lead to complete extinction, making the equilibrium resource concentration $\hat{r}$ equal to the equilibrium total concentration ρ. At $\varphi = 0$, there is no resource inflow, yielding $\hat{r} = 0$ and no production, but no outflow either, so the total replicator concentration remains at its initial value, but the association-dissociation equilibrium is reached. Typical curves of $\hat{r}$ as a function of ρ and φ are shown in Sections B.6.1 and B.6.2.

B.1.2 Coexistence of S-species and one E-species

In this subsection, we extend the set of replicator species by an E-species denoted by index 0. For the E-species, the association rate is zero ($a_0 = 0$). The corresponding set of differential equations (Eq. B.1) is extended by the equations of the E-species:

$$\begin{aligned}\dot{x}_0 &= 2b_0 y_0 - c_0 r x_0 - x_0\varphi \\ \dot{y}_0 &= -b_0 y_0 + c_0 r x_0 - y_0\varphi.\end{aligned} \tag{B.11}$$

Besides, the equation of the resource concentration changes to

$$\dot{r} = -r\sum_{i=0}^{N} c_i x_i - (r-\rho)\,\varphi, \tag{B.12}$$

where $\rho = \hat{r} + \sum_{j=0}^{N} \hat{x}_j + 2\hat{y}_j$. Based on Eq. B.11, the resource concentration at the equilibrium can be expressed as

$$\hat{r} = \frac{\varphi}{c_0}\frac{b_0+\varphi}{b_0-\varphi}, \tag{B.13}$$

showing that in the presence of an E-species, $\hat{r}$ is not affected by the S-species and the total equilibrium concentration ρ but is determined solely by the E-species and the dilution rate φ, being an increasing function of φ.

The equilibrium concentrations of the S-species are the same as in Eq. B.5, but with $\hat{r}$ being defined by Eq. B.13. Since $\hat{r}$ is independent of the equilibrium total concentration ρ, the equilibrium concentrations of the S-species do not change with ρ either. Accordingly, in the presence of an E-species, any increase in the equilibrium total concentration ρ is absorbed by the concentration of the E-species.

The equilibrium concentration of the E-species can be obtained by splitting the summation in Eq. B.12:

$$0 = -\hat{r}c_0\hat{x}_0 - \hat{r}\sum_{i=1}^{N} c_i\hat{x}_i - (\hat{r}-\rho)\varphi. \tag{B.14}$$

Solving this equation leads to

$$\hat{x}_0 = \frac{\varphi(\rho-\hat{r}) - \hat{r}\sum_{i=1}^{N} c_i\hat{x}_i}{c_0\hat{r}}, \tag{B.15}$$

from which, using that (based on the equation $\dot{y}_0 = 0$ derived from Eq. B.11) $\frac{\hat{y}_0}{\hat{x}_0} = \frac{c_0\hat{r}}{b_0+\varphi}$, we obtain

$$\hat{y}_0 = \frac{\varphi(\rho-\hat{r}) - \hat{r}\sum_{i=1}^{N} c_i\hat{x}_i}{b_0+\varphi}, \tag{B.16}$$

showing that the E-species' equilibrium concentration grows linearly with ρ. Utilizing Eqs. B.13, B.15 and B.16, it can be derived that the ratio of the double- and the single-stranded replicators of the E-species has the same form as in the case of S-species (cf. Eq. B.6):

$$\frac{\hat{y}_i}{\hat{x}_i} = \frac{c_i\hat{r}-\varphi}{2\varphi}. \tag{B.17}$$

In Eq. A.22, we saw that in the mass-regulated model $\hat{\varphi} = \lambda_0$, as an E-species can only be at equilibrium at a single outflow rate when the resource concentration of the system (and thus, the

growth rate of the E-species) is fixed. Here, substituting the equilibrium resource concentration given by Eq. B.13 in the formula of the intrinsic growth rate of the E-species (Eq. B.2 at $i = 0$), we obtain that independently from the coexisting S-species, the E-species sets the equilibrium resource concentration $\hat{r}$ to the value at which its intrinsic growth rate is

$$\hat{\lambda}_0 = \frac{-\left(b_0 + c_0 \frac{\varphi}{c_0} \frac{b_0+\varphi}{b_0-\varphi}\right) + \sqrt{b_0^2 + c_0^2 \left(\frac{\varphi}{c_0} \frac{b_0+\varphi}{b_0-\varphi}\right)^2 + 6 b_0 c_0 \frac{\varphi}{c_0} \frac{b_0+\varphi}{b_0-\varphi}}}{2} = \varphi, \tag{B.18}$$

where the simplification to the fixed outflow φ holds at any $0 < \varphi < b_0$ (which condition is also necessary to ensure that $\hat{r}$ given by Eq. B.13 is positive). This shows that at a given dilution rate φ, an E-species can only be at equilibrium at a single resource concentration, which makes the growth rate of the E-species equal to the system's constant outflow rate. In contrast, an S-species can satisfy several equilibrium conditions imposed by a given resource concentration $\hat{r}$, since its growth rate depends on its concentration (due to its concentration-dependent ratio of double- vs. single-stranded replicators). Therefore, in the presence of an E-species, the equilibrium resource concentration of the system is determined by this single species alone (see Eq. B.13), while the concentrations of the S-species are adjusted to match this $\hat{r}$ value at the given φ (see Eq. B.3).

B.2 Invasion of a new species into an established community

A new species can invade an established community if it can spread at low concentrations. Denoting the concentrations and the parameters of the invader by prime, the dynamics of its total concentration $\mu' = x' + 2y'$ can be formulated based on Eq. B.1 as

$$\frac{\mathrm{d}\mu'}{\mathrm{d}t} = c' \hat{r}_{\mathrm{res}} x' - \varphi \mu', \tag{B.19}$$

where $\hat{r}_{\mathrm{res}}$ is the equilibrium resource concentration of the resident community. The above equation holds for both sub-exponential ($a' > 0$) and exponential ($a' = 0$) invaders, since the terms describing association and dissociation always drop out from $\mathrm{d}\mu'/\mathrm{d}t$.

The criterion for successful invasion is $0 < \mathrm{d}\mu'/\mathrm{d}t$, which can be written based on Eq. B.19 as

$$\varphi < \frac{c' \hat{r}_{\mathrm{res}} x'}{\mu'} \tag{B.20}$$

or, recognizing on the right side the invader's intrinsic growth rate λ' measured at the resident system's resource concentration, as

$$\varphi < \lambda'(\hat{r}_{\mathrm{res}}). \tag{B.21}$$

Accordingly, the new species can spread in an equilibrium system from an infinitesimally small initial concentration if its initial growth rate at the current resource concentration of the system is greater than the (fixed) relative outflow of the system given by φ (which, because of the equilibrium, is equal to the growth rate of all the resident species). Note that in the mass-regulated model, where a constant resource concentration is assumed, the corresponding formula is $\hat{\varphi}_{\mathrm{res}} < \lambda'$ (Eq. A.29), where instead of the intrinsic growth rate of the invader (that is constant since $\dot{r} = 0$), the equilibrium outflow is the term that depends on the composition of the current resident system. An illustration of the invasion process in the two models is given by Fig. B.1.

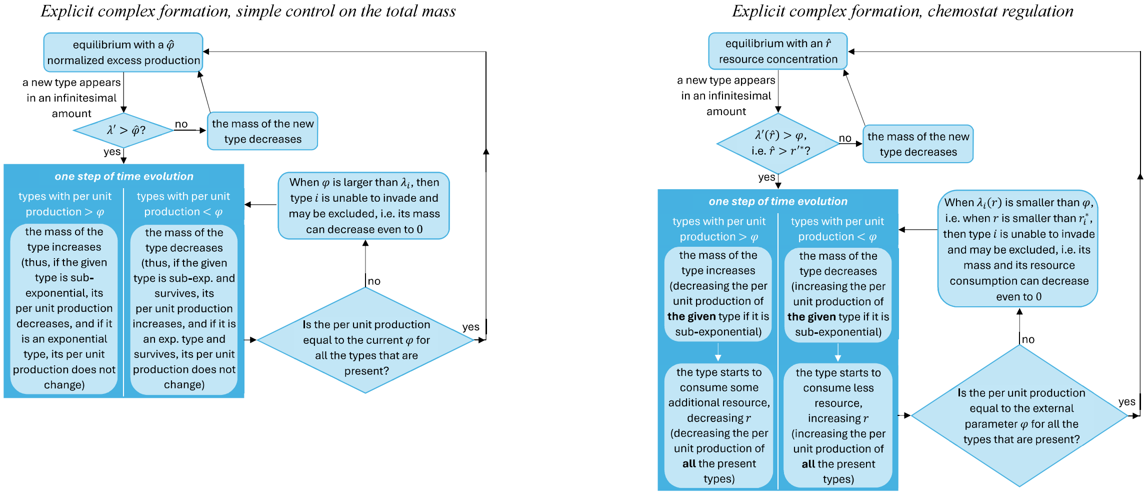

Figure B.1: Invasion of a new species at fixed external parameters in the mass-regulated model (left) and the resource-regulated model (right) of explicit complex formation. The growth rate of species i can be calculated as $\frac{rc_ix_i}{x_i+2y_i}$. This is an increasing function of the resource concentration r and a decreasing function of the ratio y_i/x_i of double- vs. single-stranded replicators. Its possible maximum at a given resource concentration r is $\lambda_i(r)$. While $\hat{\varphi} = \frac{r}{m}\sum_{j=1}^{N} c_j\hat{x}_j$ on the left and $\hat{r}$ on the right changes in every cycle, r on the left and φ on the right is a fixed parameter of the system. On the left, due to the fixed value of r, a species having a higher λ_i both grows faster at its appearance and is less vulnerable after its spread. On the right, the species that can spread in the system under the most diverse circumstances (being the most resistant against extinction due to a decrease in the resource concentration) are those having the smallest r_i^* values, but a smaller critical resource concentration does not necessarily imply a faster spread when appearing at a given $\hat{r}$ value since $\lambda_j(\hat{r})$ can be larger than $\lambda_i(\hat{r})$ even if $r_i^* < r_j^*$ (i.e., if $\lambda_j(r_i^*) < \lambda_i(r_i^*) = \varphi$ and $\lambda_i(r_j^*) > \lambda_j(r_j^*) = \varphi$). Nevertheless, just like the same λ_i value decides on the left both whether species i can appear in the system and whether it loses its ability to avoid exclusion, the same r_i^* value determines on the right the ability of species i both to invade the system and to avoid exclusion later.

An important difference compared to the mass-regulated model is that in the resource-regulated model the outflow is set as an input parameter of the model and not by the coexisting species. Thus, φ can take values at which the appearance of a given species is not possible at all. Namely, if the outflow is faster than any step of the production of new replicators, i.e. if $\varphi > b'$ or $\varphi > c'\hat{r}_{\text{res}}$, then the growth of the given species' total mass μ' cannot be faster than the outflow, i.e. λ' cannot be larger than φ and the given species can never spread in the system. At $b' \leq \varphi$ or at $c'\hat{r}_{\text{res}} \leq \varphi$, Eq. B.21 yields the condition $\varphi < 0$. Consequently, Eq. B.21 can be fulfilled by a replicator species at positive outflows only if $b' > \varphi$ and $c'\hat{r}_{\text{res}} > \varphi$. Note that these conditions are also needed to avoid negative terms in Eqs. B.5, B.6, B.10 (through the formula of Θ_1 in Eq. B.8) and B.13.

According to Eq. B.21, one can define a critical resource concentration r'^{*} above which a new species parametrized by a', b' and c' can invade an established community. Namely, using Eq. B.2, the equation $\lambda'(r'^{*}) = \varphi$ (expressing that the growth rate of the invader at its initial, infinitesimally

small concentration is the same as the growth rate of any resident species of the invaded equilibrium system) yields

$$r'^{*} = \frac{\varphi^2 + b'\varphi}{b'c' - c'\varphi} = \frac{\varphi}{c'} \cdot \frac{b' + \varphi}{b' - \varphi}. \tag{B.22}$$

Note that expressing $\hat{r}$ from Eq. B.5 at $\hat{x}_i = 0$ yields the same formula. Besides, the equilibrium resource concentration set by an E-species (Eq. B.13) is equal to the critical resource concentration of the given E-species. As a comparison, in the mass-regulated model an E-species sets the equilibrium value of the normalized production $\hat{\varphi}$ exactly to its intrinsic growth rate λ_0 measured at the given constant r of the system (see Eq. A.22).

Using the critical resource concentration of the invader, the condition of the successful invasion given by Eq. B.21 can be expressed as

$$r'^{*} < \hat{r}_{\text{res}}, \tag{B.23}$$

i.e. the new species can spread in an equilibrium system from an infinitesimally small initial concentration if its initial need of resource is smaller than the resource concentration of the invaded system. According to Eq. B.22, $r'^{*} \to \infty$ at $b' \to \varphi$, which indicates the above-mentioned impossibility of invasion at $b' = \varphi$ at any finite resource concentration.

Note that at $\hat{r} = r_i^*$, $\mathrm{d}\mu_i/\mathrm{d}t = 0$, meaning that the concentration of species i does not change in time. Thus, if the resource concentration of the system decreases to the r_i^* value of a resident S-species (corresponding to the smallest resource concentration required by the given S-species to resist the outflow at least at its infinitesimal concentration when its growth rate at the given resource concentration is maximal), then this S-species remains in the system at a constant concentration of $\mu_i \approx 0$, which practically means that it dies out. Nonetheless, due to its concentration-independent growth rate at any given amount of resources, a resident E-species can maintain a finite concentration and survive even at $\hat{r} = r_0^*$ (if the total sub-exponential replicator concentration does not reach $\rho - \hat{r}$). To sum up, as it is shown by Table B.1, the condition of spreading from an infinitesimally small concentration is given by Eq. B.23 for both S- and E-species, but resident exponential species can survive even at $\hat{r} = r_0^*$, while resident sub-exponential replicators remain in the system only if $r_i^* < \hat{r}$.

	S invader	E invader
$r^* < \hat{r}_{\text{res}}$	survive	survive
$r^* = \hat{r}_{\text{res}}$	die out	die out
$r^* > \hat{r}_{\text{res}}$	die out	die out

	S resident	E resident
$r^* < \hat{r}_{\text{new}}$	survive	cannot happen
$r^* = \hat{r}_{\text{new}}$	die out	survive
$r^* > \hat{r}_{\text{new}}$	die out	die out

Table B.1: Survival and extinction of sub-exponential (S) and exponential (E) invaders (appearing in the system at an infinitesimally small concentration) and residents (being present in a finite concentration) based on the relation between the critical resource concentration r^* of the given species and the equilibrium resource concentration before the invasion ($\hat{r}_{\text{res}}$) and after any change in the parameters or the composition of the system ($\hat{r}_{\text{new}}$).

B.3 The resource concentration cannot increase after successful invasion

In the case of a purely sub-exponential system, the quadratic equation the positive root of which defines the equilibrium resource concentration is as follows (see Eq. B.7):

$$\sum_{i=1}^{N} \frac{c_i^2(b_i-\varphi)}{2a_i\varphi}\hat{r}^2 + \left[\varphi - \sum_{i=1}^{N} \frac{c_i(b_i+\varphi)}{2a_i}\right]\hat{r} - \varphi\rho = 0. \tag{B.24}$$

As all the parameters are positive, the $(+,+,-)$ sign pattern guarantees that there is a single positive root.

In the case of a single S-species ($N = 1$) and $\rho = \hat{r}$ (note that $\rho = \hat{r} + \sum_{j=1}^{N} \hat{x}_j + 2\hat{y}_j$ in general, i.e. the setting $\rho = \hat{r}$ corresponds to that case when the concentration of the species being present is low), Eq. B.24 takes the form

$$\frac{c'^2(b'-\varphi)}{2a'\varphi}\hat{r}^2 + \left[\varphi - \frac{c'(b'+\varphi)}{2a'}\right]\hat{r} - \varphi\hat{r} = 0, \tag{B.25}$$

where prime denotes the parameters of the considered single species. This can be written at $\hat{r} > 0$ as

$$\frac{c'^2(b'-\varphi)}{2a'\varphi}\hat{r} - \frac{c'(b'+\varphi)}{2a'} = 0, \tag{B.26}$$

yielding the critical resource concentration of the given species (Eq. B.22) as the solution:

$$r'^* = \frac{\varphi}{c'} \cdot \frac{b'+\varphi}{b'-\varphi}. \tag{B.27}$$

By introducing the notations $\vartheta_1 = \frac{c'^2(b'-\varphi)}{2a'\varphi}$, $\vartheta_2 = \frac{c'(b'+\varphi)}{2a'}$ and using the notations $\Theta_1 = \sum_{j=1}^{N} \frac{c_j^2(b_j-\varphi)}{2a_j\varphi}$, $\Theta_2 = \sum_{j=1}^{N} \frac{c_j(b_j+\varphi)}{2a_j}$ defined in Eq. B.8, Eqs. B.24 and B.25 take the following form:

$$\Theta_1\hat{r}^2 + [\varphi - \Theta_2]\hat{r} - \varphi\rho = 0, \tag{B.28}$$

$$\vartheta_1\hat{r}^2 - \vartheta_2\hat{r} = 0. \tag{B.29}$$

The positive roots of these two equations are $\hat{r}_{\text{res}}$ (the resource concentration of the resident system) and r'^* (the critical resource concentration of the invader), respectively. The sum of the two equations is

$$(\Theta_1 + \vartheta_1)\hat{r}^2 + (\varphi - \Theta_2 - \vartheta_2)\hat{r} - \varphi\rho = 0, \tag{B.30}$$

the positive root of which is the $\hat{r}_{\text{new}}$ equilibrium resource concentration of the resulting system if the invasion was successful. It is easy to see that this root lies between the roots of the two parabolas defined by Eqs. B.28 and B.29: since the only positive root of Eq. B.29 (corresponding to r'^*) is smaller than the only positive root of Eq. B.28 (corresponding to $\hat{r}_{\text{res}}$) in the case of successful invasion (see Eq. B.23), the parabola described by Eq. B.28 takes negative values and the parabola described by Eq. B.29 takes positive values in the range $(r'^*, \hat{r}_{\text{res}})$, while in $[0, r'^*)$ both parabolas are negative and above $\hat{r}_{\text{res}}$ both are positive, meaning that the positive root of the sum of the two parabolas described by Eq. B.30 must fall in the range $(r'^*, \hat{r}_{\text{res}})$. Thus,

$$r'^* < \hat{r}_{\text{new}} < \hat{r}_{\text{res}}, \tag{B.31}$$

showing that the equilibrium resource concentration of a purely sub-exponential system decreases as a result of a successful invasion of an S-species.

Although the success of the invasion (i.e., the invasiveness of the invader species) depends only on b' and c' and not on a' (since r'^* given by Eq. B.22 does not depend on a'), the resource concentration in the new equilibrium forming after a successful invasion depends on a' too, so the association rate of the invader scales its competitiveness: a lower association rate (a') results in lower resource concentration ($\hat{r}_{\text{new}}$), i.e. a higher chance of excluding species from the original system. Since ϑ_1, $\vartheta_2 \sim 1/a'$, dividing a' with some positive constant results in a simple multiplication of the whole parabola described by Eq. B.29 with the same constant. Thus, when using a constant larger than 1 (i.e., when decreasing the association rate of the new species), all the positive parts of the parabola go upwards (while the roots do not change), which shifts the positive root of the summed parabola given by Eq. B.30 towards a smaller value, i.e. closer to r'^* and farther from $\hat{r}_{\text{res}}$. Accordingly, for given $\hat{r}_{\text{res}}$ and r'^*, the increase in a' increases the $\hat{r}_{\text{res}} - \hat{r}_{\text{new}}$ distance, enabling the exclusion of resident species of a larger range of critical resource concentration. As $a' \to \infty$, $\hat{r}_{\text{new}} \to \hat{r}_{\text{res}}$, while as $a' \to 0$, $\hat{r}_{\text{new}} \to r'^*$.

When a purely sub-exponential system is invaded by an exponential species ($a' = 0$), then $\hat{r}_{\text{new}} = r'^*$ (see Eq. B.13). According to Eq. B.23, such an invasion is successful if $r'^* < \hat{r}_{\text{res}}$, meaning that in this case $r'^* = \hat{r}_{\text{new}} < \hat{r}_{\text{res}}$. If the resident system contains an E-species, then an exponential invader can spread only if its critical resource concentration is smaller than that of the resident E-species, yielding again $r'^* = \hat{r}_{\text{new}} < \hat{r}_{\text{res}}$. And finally, if a sub-exponential invader fulfills the criterion of Eq. B.23 and thereby manages to invade in a system where the resource concentration is set by an E-species, then $\hat{r}_{\text{new}} = \hat{r}_{\text{res}}$ if the E-species does not go extinct with the appearance of the new species and $\hat{r}_{\text{new}} < \hat{r}_{\text{res}}$ otherwise (i.e., when the resource concentration maintained by the coexisting sub-exponential species without the exponential one decreases below the critical resource concentration of the exponential replicator). The changes in $\hat{r}$ and the coalition composition due to a successful invasion in the different scenarios are illustrated by Fig. B.2.

B.4 The largest coexisting subset of a library of replicator species

As we have seen in Section B.2, invasion will be successful in the resource-regulated model if the resource concentration of the resident community is greater than the critical resource concentration of the invader (see Eq. B.23), making the intrinsic growth rate of the invader at the resident system's resource level larger than the system's fixed outflow (see Eq. B.21). This result can be used to determine which members of a given set of replicator species (characterized by parameter triplets $\{a_i, b_i, c_i\}$) will coexist in a system with a given equilibrium total concentration ρ and dilution rate φ.

Without loss of generality, we assume that species 1 has the lowest critical resource concentration, species 2 has the second lowest, and so on, i.e. $r_1^* < r_2^* < \cdots < r_N^*$. (Note that among the species that have the same critical resource concentration, if one of them can spread in the system, then all of them can spread simultaneously.) The replicator species with the lowest critical resource concentration r_1^* is the one that can spread in the system under the most diverse circumstances. Species 1 appears in an empty system if $\rho > r_1^*$. According to Eq. B.23, the species with the second lowest critical resource concentration r_2^* can spread in the presence of the first species if $\hat{r}(\{a_1, b_1, c_1\}; \rho, \varphi) > r_2^*$. By repeating this procedure, the largest set of coexisting species can be found by arranging the replicator species in the increasing order of their critical resource concentrations, and adding the subsequent species to the coexisting set one at a time, as long as their

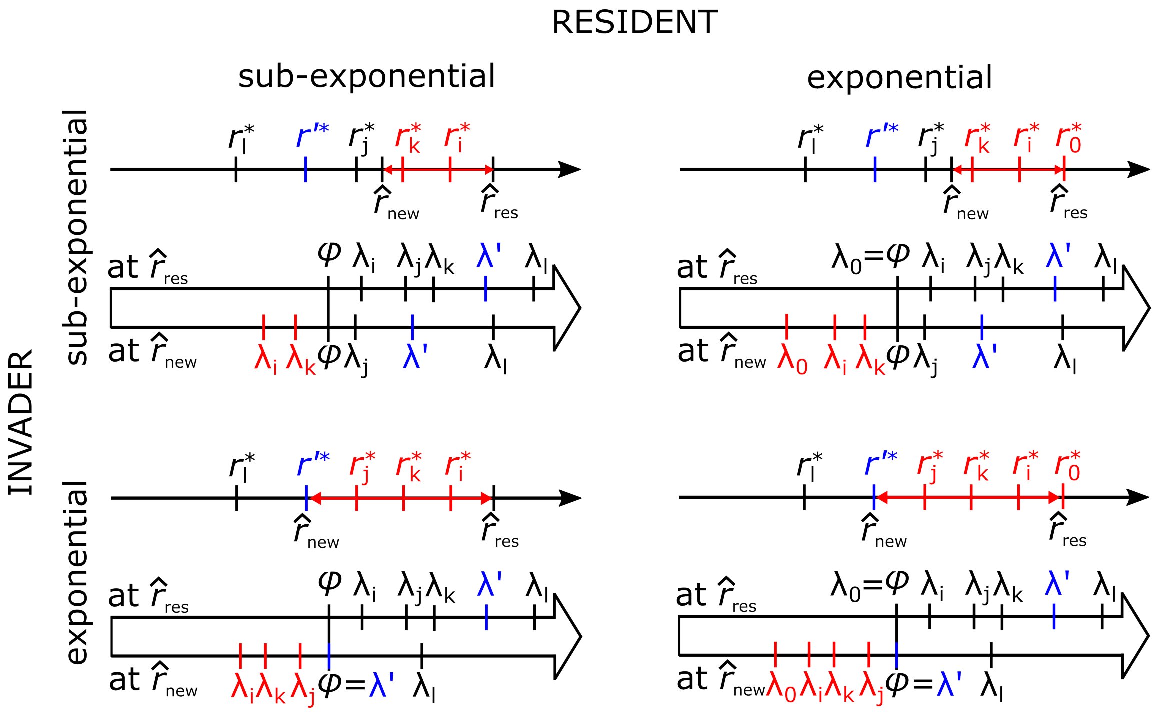

Figure B.2: Species exclusion in the resource-regulated model as a result of a successful invasion of a sub-exponential (upper row) or an exponential (lower row) replicator species into an established community of sub-exponential (left column) or sub-exponential and exponential (right column) species. In each of the four cases, the upper arrow illustrates the process from the viewpoint of the system's resource concentration and the critical resource concentrations of the different species, while the lower arrow demonstrates the changes regarding the relation between the system's dilution rate and the intrinsic growth rates of the different species. The equilibrium resource concentration of the resident and the newly established community is denoted by $\hat{r}_{\mathrm{res}}$ and $\hat{r}_{\mathrm{new}}$, respectively. The dilution rate of the system is fixed to φ during the whole process. The critical resource concentration of the invader is r'^*, while λ' is its (r-dependent) intrinsic growth rate, these are shown in blue. The critical resource concentration and the intrinsic growth rate of the replicator species that went extinct due to the invasion are marked in red, the red interval on the r-axis is the zone of exclusion. As it is exemplified by species j and k, the order of the intrinsic growth rates can change with the shift in the equilibrium resource concentration from $\hat{r}_{\mathrm{res}}$ to $\hat{r}_{\mathrm{new}}$. Note that with the exception of the bottom right panel, where the successfully invading new E-species always excludes at least the resident E-species (denoted by 0), extinction is not necessarily implied by a successful invasion. In the absence of species exclusion, $\hat{r}_{\mathrm{new}}$ is still smaller than $\hat{r}_{\mathrm{res}}$ when the original community is purely sub-exponential (left column), but $\hat{r}_{\mathrm{new}} = \hat{r}_{\mathrm{res}}$ if the resident community contains an E-species (upper right panel).

critical resource concentration meets the invasion criterion against the current equilibrium resource concentration. Since successful invasions cannot increase the equilibrium resource concentration (see Section B.3), thus the largest set of coexisting replicator species is the one that minimizes the value of $\hat{r}$. Note that if an E-species is included in the coexisting set, then $\hat{r}$ is equal to the critical resource concentration of that species, which is the largest among the coexisting species.

B.5 Ecological stability of coexisting replicators in the chemostat

This section examines how the changes of different model parameters alters the equilibrium characteristics in the resource-regulated model. For the sake of simplicity, we change only one parameter at a time, and assume such small parameter changes that do not yet cause a change in the composition of the coexisting system, i.e. do not lead to extinctions. First, Sections B.5.1 and B.5.2 present how small changes in the association rate, the dissociation rate and the replication rate affect the equilibrium value of the resource concentration and the replicator concentrations. The equilibrium total concentration ρ and the dilution rate φ are kept at a fixed value in this analysis. Then, Section B.5.3 deals with the impact of changing the external model parameters ρ and φ. The results of Sections B.5.1–B.5.3 are summarized in Table B.2. Finally, Section B.5.4 specifies the limits of the parameter changes set by the assumption of no extinction.

	$\hat{\mu}_i$	$\hat{\mu}_j$	$\hat{r}$
$a_i \uparrow$	$\downarrow$	$\uparrow$	$\uparrow$
$b_i, c_i \uparrow$	$\uparrow$	$\downarrow$	$\downarrow$
$\rho \uparrow$	$\uparrow$	$\uparrow$	$\uparrow$
$\varphi \uparrow$	$\updownarrow$	$\updownarrow$	$\uparrow$

	$\hat{\mu}_i$	$\hat{\mu}_j$	$\hat{\mu}_0$	$\hat{r}$
$a_i \uparrow$	$\downarrow$	—	$\uparrow$	—
$b_i, c_i \uparrow$	$\uparrow$	—	$\downarrow$	—
$b_0, c_0 \uparrow$	$\downarrow$	$\downarrow$	$\uparrow$	$\downarrow$
$\rho \uparrow$	—	—	$\uparrow$	—
$\varphi \uparrow$	$\updownarrow$	$\updownarrow$	$\updownarrow$	$\uparrow$

Table B.2: The direction of change in the equilibrium value of the replicator concentrations and the resource concentration as a result of increasing rate constants (top rows) or increasing external parameters (bottom rows) for purely sub-exponential systems (left table) and systems that consist of sub-exponential species and an exponential one indexed by 0 (right table). When one parameter is changed, all the others are kept constant. Notations: $\uparrow$ and $\downarrow$ denote increase and decrease, respectively; $\updownarrow$ means ambiguous change.

B.5.1 Changing the parameters of a single species in a purely sub-exponential system

As it is written in Eq. B.7, at the equilibrium of a system of S-species the resource concentration $\hat{r}$ satisfies the following equation:

$$\sum_{i=1}^{N} \frac{c_i^2(b_i-\varphi)}{2a_i\varphi}\hat{r}^2 + \left[\varphi - \sum_{i=1}^{N} \frac{c_i(b_i+\varphi)}{2a_i}\right]\hat{r} - \varphi\rho = 0. \tag{B.32}$$

Using the notations

$$\vartheta_{1,i} = \frac{c_i^2(b_i-\varphi)}{2a_i\varphi}, \quad \vartheta_{2,i} = \frac{c_i(b_i+\varphi)}{2a_i}, \tag{B.33}$$

Eq. B.32 can be rewritten as

$$\vartheta_{1,i}\hat{r}^2 - \vartheta_{2,i}\hat{r} + \sum_{j=1,j\neq i}^{N} \vartheta_{1,j}\hat{r}^2 + \left[\varphi - \sum_{j=1,j\neq i}^{N} \vartheta_{2,j}\right]\hat{r} - \varphi\rho = 0, \tag{B.34}$$

which can be considered as a sum of two parabolas that are given by the equations $\vartheta_{1,i}\hat{r}^2 - \vartheta_{2,i}\hat{r} = 0$ and $\sum_{j=1,j\neq i}^{N} \vartheta_{1,j}\hat{r}^2 + \left[\varphi - \sum_{j=1,j\neq i}^{N} \vartheta_{2,j}\right]\hat{r} - \varphi\rho = 0$. The value of $\hat{r}$ is given by the positive root of the summed parabola, while the positive root of the first parabola corresponds to r_i^* and the positive root of the second parabola (being larger than r_i^*, since otherwise species i could not be present, see Eq. B.31) corresponds to the equilibrium value of the resource concentration in the absence of species i. According to Section B.3, as the association rate a_i decreases (thereby stretching the first parabola), the root of the summed parabola decreases. So we can conclude that an increase in a_i leads to an increase in $\hat{r}$.

Changing the dissociation rate b_i or the replication rate c_i changes the positive root of the first parabola $\vartheta_{1,i}\hat{r}^2 - \vartheta_{2,i}\hat{r} = 0$ given by the critical resource concentration $r_i^* = \frac{\varphi}{c_i} \cdot \frac{b_i+\varphi}{b_i-\varphi}$ (Eq. B.27), while the positive root of the second parabola $\sum_{j=1,j\neq i}^{N} \vartheta_{1,j}\hat{r}^2 + \left[\varphi - \sum_{j=1,j\neq i}^{N} \vartheta_{2,j}\right]\hat{r} - \varphi\rho = 0$ remains the same (somewhere above r_i^*). An increase in b_i or c_i makes r_i^* smaller, which, by shrinking the range at which the first parabola takes negative values while leaving the second parabola unaltered, shrinks the range at which the summed parabola takes negative values, i.e. shifts the positive root of the summed parabola to a smaller value. Consequently, an increase in b_i or c_i leads to a decrease in $\hat{r}$.

But what happens with the species concentrations due to a change in $\hat{r}$? Using Eq. B.5, the total concentration of a given S-species can be written at equilibrium as

$$\hat{\mu}_i = \hat{x}_i + 2\hat{y}_i = \frac{c_i\hat{r}}{a_i} \cdot \frac{-\varphi^2 - (b_i + c_i\hat{r})\varphi + b_i c_i \hat{r}}{2\varphi^2} = \frac{c_i^2\hat{r}^2(b_i - \varphi) - c_i\hat{r}(b_i + \varphi)\varphi}{2a_i\varphi^2}, \tag{B.35}$$

which is the same as Eq. A.37 with φ and $\hat{r}$ substituting $\hat{\varphi}$ and r, respectively. With the rearrangement of Eq. B.35, it can be shown that besides Eq. B.32, the equation

$$\frac{c_i^2(b_i - \varphi)}{2a_i\varphi}\hat{r}^2 - \frac{c_i(b_i + \varphi)}{2a_i}\hat{r} - \varphi\hat{\mu}_i = 0 \tag{B.36}$$

is also true for $\hat{r}$ at any species index i. With the notations given by Eq. B.33, Eq. B.36 can be written in a form similar to Eq. B.9 (note that $\rho = \hat{r} + \sum_{j=1}^{N} \hat{\mu}_j$, and thus $\varphi\hat{r} - \varphi\rho = -\varphi \sum_{j=1}^{N} \hat{\mu}_j$), namely as

$$\vartheta_{1,i}\hat{r}^2 - \vartheta_{2,i}\hat{r} - \varphi\hat{\mu}_i = 0. \tag{B.37}$$

We know that $\hat{r}$ is a solution of Eq. B.37 for any species index i even after changing a parameter of species i. For any $j \neq i$, the change in a_i, b_i or c_i does not change $\vartheta_{1,j}$ and $\vartheta_{2,j}$, meaning that for $j \neq i$, only $\hat{r}$ and $\hat{\mu}_j$ are affected in Eq. B.37 by the change of a parameter of species i. To satisfy Eq. B.37 at a smaller $\hat{r}$ value and the same $\vartheta_{1,j}$, $\vartheta_{2,j}$ values, $\hat{\mu}_j$ has to be smaller, meaning that a decrease in $\hat{r}$ caused by the change of a_i, b_i or c_i decreases $\hat{\mu}_j$ for any $j \neq i$. And since $\rho = \hat{r} + \sum_{j=1}^{N} \hat{\mu}_j$ is always true, the decrease in the total concentration of all the other species and in the resource concentration implies an increase in $\hat{\mu}_i$.

To sum up, as it is shown by the left panel of Table B.2, in the absence of any E-species, an increase in a_i leads to a decrease in $\hat{\mu}_i$ and an increase in $\hat{r}$ and in $\hat{\mu}_j$ for any $j \neq i$, while an increase in b_i or c_i yields an increase in $\hat{\mu}_i$ and a decrease in $\hat{r}$ and $\hat{\mu}_j$ for any $j \neq i$.

B.5.2 Changing the parameters of a single species in a system that contains an E-species

In the presence of an E-species (having an association rate $a_0 = 0$), the equilibrium resource concentration is determined by r_0^* (see Eqs. B.13 and B.22), and therefore, changing the parameters of any S-species i has no impact on $\hat{r}$. Accordingly, using that the total equilibrium concentration of any S-species is given by Eq. B.35, it is easy to see that changing a_i, b_i or c_i affects only $\hat{\mu}_i$ and does not influence $\hat{\mu}_j$ for any $j \neq i$. Nevertheless, the equilibrium concentration of the E-species does not remain unaltered when a parameter of an S-species changes: to keep the total concentration of the system at the fixed equilibrium value of ρ, an increase in $\hat{\mu}_i$ is compensated by a decrease in $\hat{\mu}_0$ and a decrease in $\hat{\mu}_i$ yields an increase in $\hat{\mu}_0$.

The dependence of $\hat{\mu}_i$ on a_i is obvious from Eq. B.35: larger values of a_i yield smaller values of $\hat{\mu}_i$. The impact of changing b_i or c_i on $\hat{\mu}_i$ can be seen from Eq. B.37, interpreting this formula as the equation of the vertically shifted parabola obtained by decreasing with $\varphi\hat{\mu}_i$ all the points of the parabola defined as $\vartheta_{1,i}\hat{r}^2 - \vartheta_{2,i}\hat{r} = 0$. The positive root of the non-shifted parabola is r_i^* (see Eq. B.29), which is a decreasing function of b_i and c_i. The root of the shifted parabola given by Eq. B.37 is the whole system's $\hat{r}$ value, which is set by the E-species to r_0^*. Thus, to keep the root of the shifted parabola unaltered, in the case of increasing b_i or c_i and thereby decreasing r_i^*, the downward shift of $\varphi\hat{\mu}_i$ must be increased, which can be realized by an increase in $\hat{\mu}_i$.

But what happens if we change the dissociation or the replication rate of the E-species instead of an S-species? The growth of both b_0 and c_0 decreases r_0^* (see Eq. B.13). Using again Eq. B.37, one can see that for an unaltered r_i^* root of the parabola $\vartheta_{1,i}\hat{r}^2 - \vartheta_{2,i}\hat{r} = 0$ and a decreased r_0^* root of the shifted parabola $\vartheta_{1,i}\hat{r}^2 - \vartheta_{2,i}\hat{r} - \varphi\hat{\mu}_i = 0$, $\hat{\mu}_i$ must be decreased, meaning that the decrease in r_0^* and thereby in the equilibrium resource concentration of the system must imply a decrease in $\hat{\mu}_i$ for any $i \neq 0$. In the meantime, $\hat{\mu}_0 = \rho - \hat{r} - \sum_{i=1}^{N} \hat{\mu}_i = \rho - r_0^* - \sum_{i=1}^{N} \hat{\mu}_i$ increases.

To sum up, as it is shown by the right panel of Table B.2, in the presence of an E-species indexed by 0, an increase in the association rate a_i of any S-species of $0 < i$ yields a decrease in $\hat{\mu}_i$, an increase in $\hat{\mu}_0$ and no change in $\hat{r}$ and in $\hat{\mu}_j$ for any $0 < j \neq i$, whereas an increase in b_i or c_i of any S-species of $0 < i$ leads to an increase in $\hat{\mu}_i$, a decrease in $\hat{\mu}_0$ and no change in $\hat{r}$ and in $\hat{\mu}_j$ for any $0 < j \neq i$. Besides, an increase in the parameters b_0 or c_0 of the E-species yields an increase in $\hat{\mu}_0$ and a decrease in $\hat{r}$ and $\hat{\mu}_i$ for any $0 < i$.

B.5.3 Changing the external parameters

The bottom rows of Table B.2 summarize the effect of small changes in the external parameters ρ and φ on the equilibrium of a system consisting of only sub-exponential species (left panel) and sub-exponential species with an exponential one (right panel). The equilibrium concentration of a given S-species can be calculated according to Eq. B.35, and the equilibrium concentration of an E-species can be expressed as $\hat{\mu}_0 = \rho - \hat{r} - \sum_{i=1}^{N} \hat{\mu}_i$. The equilibrium value of the resource concentration is an increasing function of ρ in purely sub-exponential systems (see Eq. B.10), and is independent of ρ if an E-species is present (see Eq. B.13). Thus, using Eq. B.35, we can conclude that in a purely sub-exponential system, the equilibrium concentration of all species increases as the equilibrium total concentration ρ increases. If an E-species is also present, any increase in the equilibrium total concentration will be 'absorbed' by the $\hat{\mu}_0$ concentration of the E-species, while the equilibrium concentration of the resource and all the S-species remains unchanged. The effects of changes in the dilution rate φ are less general. While an increase in φ increases the equilibrium resource concentration $\hat{r}$ (see Eqs. B.10 and B.13), the change in the concentration of the different

species is unambiguous, depending on the actual set of rate constants.

B.5.4 The 'critical' parameter values that lead to the first extinction in a given coalition

In Sections B.5.1–B.5.3, we have assumed that the perturbation of the parameters is not large enough to change the set of coexisting species. As the strength of the perturbation increases, there will be a 'critical' value for the external parameters (ρ^*, φ^*) and for each rate constant (a_i^*, b_i^*, c_i^*) at which the first extinction (not necessarily of species i) occurs. In most of the cases, there is no simple analytical form for these critical values, but they can be calculated by an exhaustive search.

In a purely sub-exponential system, the equilibrium concentration $\hat{\mu}_i$ is given by Eq. B.35 for any species i. Thus, e.g. the critical association rate of a given species i can be obtained by calculating for each present species j the association rate a_i corresponding to zero equilibrium concentration $\hat{\mu}_j$. Among the obtained values, the one that is the closest to the original association rate of species i is the 'critical' association rate a_i^* of species i at which the first extinction occurs. Another option is to find a_i^* as that setting of a_i at which the equilibrium resource concentration $\hat{r}$ of the system (given by Eq. B.10) first becomes equal to the critical resource concentration $r_j^* = \frac{\varphi}{c_j} \cdot \frac{b_j+\varphi}{b_j-\varphi}$ (Eq. B.27) of any present species j. The b_i^*, c_i^* and φ^* values can be determined the same way. However, for the 'critical' value of the equilibrium total concentration ρ, there is a simple analytical formula. According to Eq. B.9, the condition $\hat{r}(\rho^*) = \tilde{r}$ with $\tilde{r} = \max r_j^*$ yields the equation $\Theta_1\tilde{r}^2 + [\varphi - \Theta_2]\,\tilde{r} - \varphi\rho^* = 0$, from which ρ^* can be expressed as

$$\rho^* = \frac{\Theta_1\tilde{r}^2 + [\varphi - \Theta_2]\,\tilde{r}}{\varphi}, \tag{B.38}$$

where the Θ values can be calculated from Eq. B.8. (The same formula for ρ^* can be obtained by searching for the largest equilibrium total concentration at which the equilibrium concentration $\hat{\mu}_i$ of any present species i goes to zero.) Note that ρ^* is also the smallest equilibrium total concentration at which a species characterized by a critical resource concentration of $\tilde{r}$ can enter the system as ρ increases. Therefore, it does not matter whether the constants of the species in question are included in the calculation of the Θ values (according to Eq. B.29, their contribution to ρ^* is zero). Nevertheless, it is important to note that ρ^* refers to a given set of species, so the Θ parameters must be recalculated after each change in the composition of the coalition.

In the presence of an E-species indexed by 0, the equilibrium resource concentration $\hat{r}$ is fixed by the E-species to its critical resource concentration r_0^* (see Eq. B.13), which is the largest critical resource concentration among the coexisting species. In this case, the equilibrium concentration $\hat{\mu}_i$ of an S-species i (given by Eq. B.35) can not be set to 0 by tuning its association rate $a_i > 0$. Here $a_i^* = 0$ for any $i \neq 0$, i.e. the first extinction due to the change in a_i occurs when species i becomes exponential: at this point, since $r_0^* > r_i^*$ for any $i \neq 0$, the original exponential species denoted by 0 will be excluded.

The adjustment of the dissociation rate b_i or the replication rate c_i of any S-species i can lead to the extinction of either the given S-species itself or the E-species of the coalition. When decreasing b_i or c_i, r_i^* increases and species i dies out ($\hat{\mu}_i$ in Eq. B.35 becomes 0) at $r_i^* = \hat{r}$, i.e. at $r_i^* = r_0^*$. The corresponding critical rate constants are $b_i^* = \varphi\frac{c_i(b_0+\varphi)+c_0(b_0-\varphi)}{c_i(b_0+\varphi)-c_0(b_0-\varphi)}$ and $c_i^* = c_0\frac{b_0-\varphi}{b_0+\varphi}\frac{b_i+\varphi}{b_i-\varphi}$. When increasing b_i or c_i, the E-species dies out ($\hat{\mu}_0 = 0$) at that b_i^* or c_i^* value for which $\rho - \sum_{i=1}^{N}\hat{\mu}_i = r_0^*$, i.e. when the equilibrium resource concentration maintained by the given set of S-species (indexed

by $i = 1, 2 \dots N$) in the absence of the E-species (which decreases with the decline of r_i^*, and can also be calculated according to Eq. B.10) reaches the critical resource concentration of the E-species. Similarly, when decreasing the dissociation rate or the replication rate of the E-species, the E-species dies out ($\hat{\mu}_0 = 0$) at that b_0^* or c_0^* value for which $r_0^* = \rho - \sum_{i=1}^{N} \hat{\mu}_i$, i.e. when the increasing critical resource concentration of the E-species reaches the equilibrium resource concentration maintained by the given set of S-species in the absence of the E-species. On the other hand, increasing b_0 or c_0 leads to the extinction of an S-species ($\hat{\mu}_i = 0$) when the decreasing equilibrium resource concentration $\hat{r}$ set by the E-species reaches the critical resource concentration r_i^* of the given S-species, i.e. at $r_0^* = r_i^*$, yielding $b_0^* = \min\limits_{1 \le i \le N} \varphi \frac{c_0(b_i+\varphi)+c_i(b_i-\varphi)}{c_0(b_i+\varphi)-c_i(b_i-\varphi)}$ and $c_0^* = \min\limits_{1 \le i \le N} c_i \frac{b_i-\varphi}{b_i+\varphi} \frac{b_0+\varphi}{b_0-\varphi}$.

Regarding the dilution rate φ, the 'critical' value at which the first extinction occurs can be calculated in the presence of an E-species the same way as in a purely sub-exponential system: one can find φ^* the most easily by tuning φ until the equilibrium concentration of any species (either sub-exponential or exponential) first becomes 0. (Note that if an S-species i is the one that dies out at φ^*, then $r_i^* = r_0^*$ at this dilution rate, while if the 'critical' outflow belongs to the extinction of the E-species, then r_0^* becomes equal to $\rho - \sum_{i=1}^{N} \hat{\mu}_i$ at φ^*, where all the critical resource concentrations and the equilibrium resource concentrations are changing with φ.) However, when changing the equilibrium total concentration ρ, then it is clear that the first species to go extinct is the exponential one since in the presence of an E-species the equilibrium resource concentration and the equilibrium concentration of any S-species are independent of ρ, and thus, a decrease in the equilibrium total concentration will decrease the concentration of the E-species only. This is consistent with the extinction order determined by the critical resource concentrations, as the E-species has highest critical resource concentration of all the coexisting species. The extinction of the E-species ($\hat{\mu}_0 = 0$) happens when the equilibrium total concentration reduces to $\rho^* = r_0^* + \sum_{i=1}^{N} \hat{\mu}_i$.

B.6 The effect of changes in the external parameters ρ and φ on the composition of coexisting species

While Section B.5 analyzed the effect of such small changes in ρ and φ that did not modify the coalition composition, here we study the effect of larger changes in the external parameters on the diversity-maintaining ability of the system. First, Section B.6.1 analyzes the impact of changing the equilibrium total concentration ρ, and then Section B.6.2 describes the effect of changing the dilution rate φ.

B.6.1 The effect of changing ρ

As already discussed in Section B.1.1, in the case of a purely sub-exponential system, the change in the equilibrium total concentration ρ affects the equilibrium resource concentration, i.e. an increase in ρ increases $\hat{r}$ (see Eq. B.10). Since successful invasion requires the critical resource concentration of the invader (which does not depend on ρ, see Eq. B.22) to be smaller than $\hat{r}$ of the resident community (see Eq. B.23), increasing the equilibrium total concentration ρ increases the diversity-maintaining ability at a given value of φ. In other words, decreasing ρ causes a gradual loss of species, as shown in Fig. B.3A, top left. The species that dies out first from the purely sub-exponential system as ρ decreases is the one with the highest critical resource concentration, since this is the first one that exceeds the decreasing resource concentration of the system. As a simple consequence, the order of extinction of different S-species as ρ decreases is determined by the critical resource concentrations (calculated at the fixed φ dilution rate of the system): the smaller

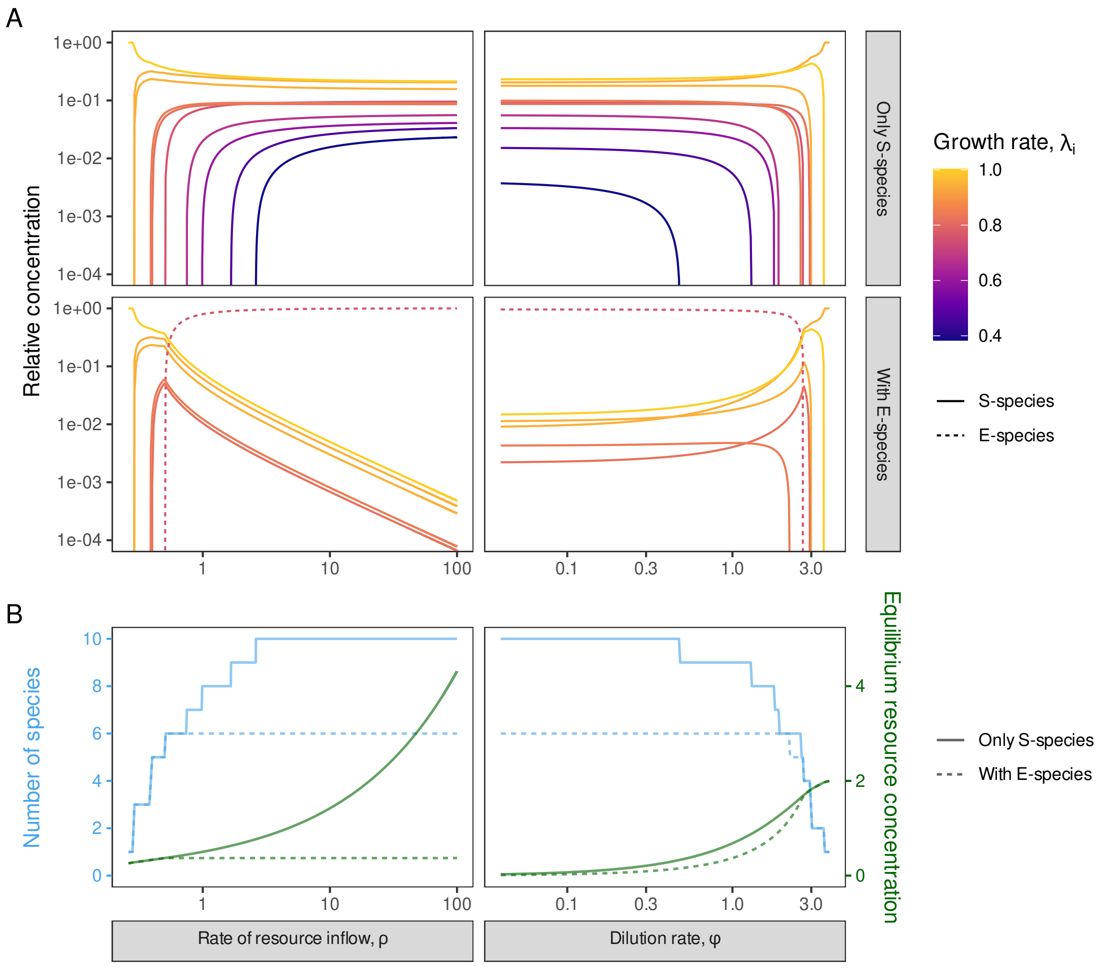

Figure B.3: Behavior of the system as a function of the equilibrium total concentration (ρ; left column) and as a function of the dilution rate (φ; right column). Panel A, top row: equilibrium relative species concentrations when the system consists of S-species only. Panel A, bottom row: equilibrium relative species concentrations when the system consists of one exponential and nine sub-exponential species, the E-species marked by the dashed line. Panel B: the resource concentration at equilibrium (green) and the number of coexisting species (blue); solid/dashed lines correspond to pure sub-exponential and exponential+sub-exponential systems. In the left column where ρ is varied, we fixed $\varphi = 1$; in the right column where φ is varied, we set $\rho = 2$. In all of these numerical simulations, we used the ten-member set of replicator species sampled from the parameter space defined in Table 1 of the main text.

the critical resource concentration r_i^* of a species, the lower the equilibrium total concentration ρ^* at which it will go extinct (Eq. B.38). Note that in the mass-regulated model, the extinctions due to the decrease of the target replicator concentration m follows the increasing order of the λ_i intrinsic growth rates (calculated at the fixed r resource concentration of the system), where λ_i can be interpreted as the critical value of the normalized production, i.e. that $\hat{\varphi}$ value below which species i can invade an established community (while r_i^* corresponds to that $\hat{r}$ value above which species i can invade an established community). In addition to the critical resource concentration which is an exact measure, the order of equilibrium concentrations ($\hat{\mu}_i$) at high equilibrium total concentration (ρ) could serve as a good proxy for the order of extinction of species: the higher the concentration, the later the extinction, see Fig. B.3A, top left.

The presence of an E-species significantly changes the behavior of the system. While an increase in ρ increases $\hat{r}$ and all the equilibrium concentrations in a purely sub-exponential system (see Eqs. B.10 and B.35), the E-species fixes the concentration of the resource (see Eq. B.13) and thereby the concentration of all the present S-species by 'absorbing' any increase in the equilibrium total concentration. Thus, the E-species dominates the system at high values of ρ, see Fig. B.3A, bottom left. Since in the presence of an E-species the equilibrium concentration of any S-species is independent of ρ, a decrease in the equilibrium total concentration ρ will decrease the concentration of the E-species only, so it will die out first. This is consistent with the extinction order determined by the critical resource concentrations, as the E-species has highest critical resource concentration of all the coexisting species (see Fig. B.2). As described in Section B.5.4, the extinction of the E-species happens when $\rho - \hat{r}$ reduces to the sum of the equilibrium concentrations of all the S-species, which are given by Eq. B.35 at $\hat{r} = r_0^*$.

Figure B.3 (B, left) shows a typical $\hat{r}(\rho)$ curve (dark green) for a purely sub-exponential system (solid line) and for a system of sub-exponential species and an exponential species (dashed line). This panel also shows the number of coexisting species (light blue) as a function of ρ. At $\rho = 0$, $\hat{r} = 0$ and no species can survive. In the purely sub-exponential system, the slope of the increase in the equilibrium resource concentration as a function of ρ increases with each new species entered, so the increasing number of species makes it more difficult for new species to enter into the system. The appearance of an E-species prevents other species of the 10-member species set from entering the system at any higher ρ values, since the critical resource concentration of the non-present species of this set is higher than that of the E-species (otherwise they would appear at a smaller ρ^* than the E-species). Nevertheless, it is important to note that other species (either sub-exponential or exponential) that have lower critical resource concentrations than the E-species can enter the system even in the presence of the E-species. A successful exponential invader replaces the existing E-species, while a new sub-exponential species may not exclude the resident exponential replicator (see Fig. B.2).

B.6.2 The effect of changing φ

Just like the equilibrium total concentration ρ, the dilution rate φ also affects the diversity-maintaining ability of the system. If the outflow (characterized by φ) is faster than any step of the production of new replicators, i.e. either the dissociation (described by b) or the replication (characterized by $c\hat{r}$), then survival is not possible (see Section B.2). Thus, large values of φ lead to complete extinction, setting $\hat{r}$ equal to the equilibrium total concentration $\rho = \hat{r} + \sum_{j=1}^{N} \hat{\mu}_j$. However, a decrease in φ makes the critical resource concentrations given by Eq. B.22 smaller, starting to enable the survival of replicator species at the $\hat{r}$ values achievable at the given ρ. Thus,

analogously to the mass-regulated model where decreasing resource availability promotes coexistence (see the right column of Fig. 2 in the main text), a decrease in the dilution rate φ (resulting in reduced resource inflow) typically increases the number of coexisting species when ρ is fixed. Nevertheless, the dependence of the diversity on φ is non-monotonic: since in the invasion criterion given by Eq. B.23 both the critical resource concentrations (Eq. B.22) and the equilibrium resource concentration (Eqs. B.10 and B.13) decrease towards lower dilution rates, a decrease in φ can both facilitate the invasion of a species or lead to its extinction.

A typical behavior of a purely sub-exponential system as a function of the dilution rate φ is shown by Fig. B.3A, top right. Compared to the behavior as a function of the equilibrium total concentration ρ, the dependence on the dilution rate φ is more complex, since the critical resource concentration of any species changes with φ while being independent of ρ. Whereas at a constant dilution rate the critical resource concentration of each species and thereby their order are fixed and determines the order of invasion (or extinction) towards larger (or smaller) values of ρ, the adjustment of φ can change the order of critical resource concentrations and thus the order of invasion or extinction. (Note that similarly, when the system's resource concentration r is adjusted in the mass-regulated model, the order of invasion or extinction is not determined by a single order of the intrinsic growth rates since they depend on r.)

As a sub-exponential, an exponential species can enter the system when the current dilution rate φ makes the resource concentration of the equilibrium system larger than the critical resource concentration of the invader in question (see Eq. B.23). An invading E-species locks the equilibrium resource concentration at its critical resource concentration (see Eqs. B.13 and B.22), which is an increasing function of φ. However, while the setting of $\hat{r}$ to r_0^* locks the community composition in the case of changing ρ, the change in φ enables further exclusions or invasions even after the appearance of the E-species since both the equilibrium resource concentration set by the E-species and the critical resource concentration of the possible invaders are φ-dependent. The bottom right panel of Fig. B.3A shows the equilibrium species concentrations as a function of dilution rate when an E-species is present in the examined 10-member species set. In this case, after the invasion of the E-species, one S-species can enter during the decrease of φ. The right panel of Fig. B.3B shows the equilibrium resource concentration and the number of coexisting species for the pure sub-exponential system and the system with one exponential species.

Appendix C: The phenomenological model

In this section, we analyze the dynamics of the phenomenological model of sub-exponential and exponential replicators in the context of the mass-regulated model, with a special emphasis on coexistence. The simplicity of the phenomenological model notwithstanding, it behaves similarity to the mechanistic mass-regulated model of Eq. 2 in the main text in the high concentration regime, and from the viewpoint of the impacts of the presence of an E-species. However, due to its diverging growth rates, the diversity of S-species produced by the phenomenological model turns out to be artifactual.

C.1 Dynamics of the system

Here we formulate the phenomenological model of sub-exponential (and exponential) replicators. A key concept is the *growth order* of replicator species i, denoted p_i: it is the power of the replicator concentration z_i to which concentration growth is proportional. For S-species, $0 < p_i < 1$, while $p_i = 1$ corresponds to E-species. The dynamics governing the system of species $i = 1, 2, \dots, N$ are then given by

$$\dot{z}_i = k_i z_i^{p_i} - \varphi z_i, \tag{C.1}$$

where k_i is a kinetic rate, φ is the normalized production and the dot denotes differentiation with respect to time (so $\dot{z}_i = \mathrm{d}z_i/\mathrm{d}t$). By setting $\varphi = (\sum_j k_j z_j^{p_j})/m$ where $m = \sum_j \hat{z}_j$ is the target replicator concentration, we introduce mass-regulation into the model in the sense of Section A.1.

C.2 Coexistence of different replicator species in the phenomenological model

In general, the steady state of the system described by Eq. C.1 cannot be solved analytically, but in some simple cases (e.g. when all growth orders are equal) the analytical solution is possible. In the following, we present results for the equilibrium of purely sub-exponential systems and systems containing both sub-exponential and exponential replicators.

C.2.1 Coexistence of S-species

As the growth rate of any S-species can be arbitrarily high when its concentration is low, therefore the diversity-maintaining ability of a sub-exponential replicator system is (theoretically) infinite in the phenomenological model, and there is generally no obstacle to the coexistence of many S-species at any finite, non-zero target replicator concentration m. As an example, Fig. C.1A shows the concentration of five sub-exponential replicators with different growth orders and equal rate constants as the function of the target replicator concentration m. Since the growth rate is proportional to $z_i^{-(1-p_i)}$, at $m \to 0$ only the species with the lowest growth order will survive. Besides, at the low m regime, the lower the growth order p_i, the higher the equilibrium concentration $\hat{z}_i$. At high total concentrations, the situation is reversed, i.e. the species with the highest growth order has the highest concentration. Note that as m increases, the concentration of the species with the highest growth order can be very high compared to the others (regardless of the rate constants), but no S-species gets excluded.

The most important and most common situation for S-species is when all their growth orders are $p = 1/2$, differing only in their rate constants k_i. In this case, the equilibrium concentration of

species i can be calculated analytically:

$$\hat{z}_i = m \frac{\prod_{j=i}^{N-1} K_j}{1 + \sum_{j=1}^{N-1} \prod_{\ell=1}^{j} K_{N-\ell}}, \tag{C.2}$$

where $K_i = \left(\frac{k_i}{k_{i+1}}\right)^{\frac{1}{1-p}}$. Accordingly, the equilibrium concentration of each species grows linearly with the target replicator concentration m. Thus, the relative equilibrium concentrations of the different species are independent of m, and the species with highest rate constant has the highest equilibrium concentration, see Fig. C.1B. The ratio between the equilibrium concentrations of replicators with the highest (k_1) and the second highest (k_2) rate constants is $\frac{\hat{z}_1}{\hat{z}_2} = K_1 = \left(\frac{k_1}{k_2}\right)^{\frac{1}{1-p}}$, which can be arbitrarily high as $p \to 1$ — this is the "precursor" of competitive exclusion.

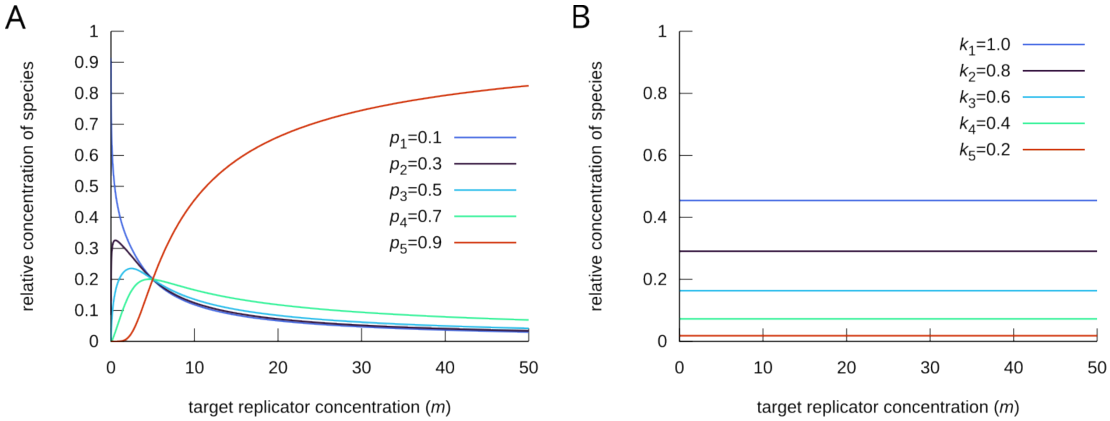

Figure C.1: Relative concentrations of 5 S-species (indexed by $i = 1, 2, \ldots, 5$) at the equilibrium of the phenomenological model as a function of the target replicator concentration. Panel A: different growth orders ($p_i = 0.1 + 0.2(i-1)$) and equal rate constants ($k_i = 1$). Panel B: equal growth orders ($p_i = 0.5$) and different rate constants ($k_i = 1 - 0.2(i-1)$).

C.2.2 Coexistence of S-species and one E-species

Assuming different S-species and an E-species, the results are quantitatively similar to those obtained from the mechanistic model: below a critical target replicator concentration, the exponential species cannot invade, the system is purely sub-exponential, see Fig. C.2A. Just like in the mechanistic model, the successful invasion of the E-species 'locks in' the concentrations of all S-species in the phenomenological model too, and these concentrations remain independent of further increases in the total concentration, see Fig. C.2B. Consequently, the concentrations of the S-species relative to each other depend on the concentration at which the E-species invades the system. Figure C.2 shows a case where due to the identical kinetic rates, the concentrations of the different S-species are the same after the invasion of the E-species.

The linear growth of the E-species with increasing m and the freezing of the concentration of S-species over a critical value of m can be proved. Let us assume arbitrary number of sub-exponential

species (their concentrations denoted by z_i, $i = 1, 2, ..., N$) and a single exponential one (z_0). The corresponding dynamics can be formulated as:

$$\begin{aligned} \dot{z}_i &= k_i z_i^{p_i} - \varphi z_i \\ \dot{z}_0 &= k_0 z_0 - \varphi z_0. \end{aligned} \tag{C.3}$$

If $z_0 > 0$, i.e. the E-species is present in the system, then in steady state ($\dot{z}_0 = 0$) the normalized production is determined solely by this species: $\hat{\varphi} = k_0$. Plugging this value into Eq. C.3, we get the steady state concentrations of S-species: $\hat{z}_i = \left(\frac{k_i}{k_0}\right)^{\frac{1}{1-p_i}}$, which are independent of m. Because of the constraint on the total concentration, the concentration of the E-species grows linearly with the target replicator concentration: $\hat{z}_0 = m - \sum_{i=1}^{N} \hat{z}_i = m - \sum_{i=1}^{N} \left(\frac{k_i}{k_0}\right)^{\frac{1}{1-p_i}}$.

In the general case, we cannot derive an analytical formula for the critical m at which the E-species can enter. But for a single S-species (with growth order p and rate constant k_1) and an E-species (with rate constant k_0), the critical concentration can easily be derived (see Section C.3.2): $m^* = \left(\frac{k_1}{k_0}\right)^{\frac{1}{1-p}}$. According to our numerical experiences, increasing the (highest) growth order of S-species and/or decreasing the rate constant of the E-species leads to an increase of the critical target replicator concentration m^*. Therefore, as in the mechanistic model, the total concentration can act as a switch between the sub-exponential and the exponential regime. Among different exponential species, the competitive exclusion remains valid (see Section C.3.4): the E-species with the highest rate constant excludes the others.

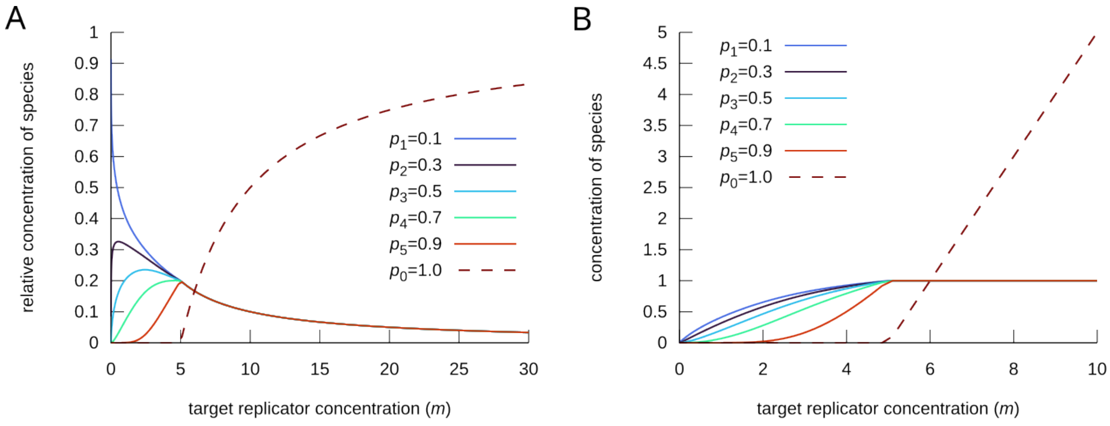

Figure C.2: Relative concentrations (panel A) and absolute concentrations (panel B) of sub-exponential (solid lines) and exponential (dashed line) species at the equilibrium of the phenomenological model as a function of the target replicator concentration m. Parameters for S-species are the same as in Fig. C.1A: $p_i = 0.1 + 0.2(i - 1)$, $k_i = 1$, $i = 1, 2, ..., 5$; parameters for the E-species: $p_0 = 1.0$, $k_0 = 1$.

C.3 Invasion analysis in the phenomenological model

In this section, we discuss the four different invasion scenarios in the context of the phenomenological model, with the simplification that the resident community consists of one species. This simplification allows for analytical tractability. In each case, the concentration of the established community and the invader is denoted by z and ζ, respectively. We assume that the invader species has a low initial concentration ϵ, so initially $z = m - \epsilon$ and $\zeta = \epsilon$. Note that the below-described results are the same if we assume that the invader comes from outside the system by setting initially z to m and ζ to ϵ. The growth order is p for the established community and q for the invader. The rate constant is k for the established community and κ for the invader.

C.3.1 Invasion of an S-species into an established sub-exponential community

The sub-exponential invader with growth order $0 < q < 1$ can grow in the established sub-exponential community (of growth order $0 < p < 1$) if $\dot{\zeta} > 0$, i.e. if

$$\kappa\epsilon^q - \epsilon/m\,[k(m-\epsilon)^p + \kappa\epsilon^q] > 0. \tag{C.4}$$

Since the initial concentration of the invader is small compared to m, the following approximation can be applied: $(m-\epsilon)^p \approx m^p - pm^{p-1}\epsilon$. After some simple rearrangement, we get

$$\frac{\kappa}{k} \cdot \frac{m-\epsilon}{m-p\epsilon} \cdot m^{1-p} > \epsilon^{1-q}. \tag{C.5}$$

This is true at any positive k, κ, p and q because $\lim_{\epsilon\to 0}\epsilon^{1-q} = 0$. Consequently, in the phenomenological model an S-species can always spread in an established sub-exponential community regardless of the growth orders and the rate constants, enabling the coexistence of an unlimited number of S-species (while in the mechanistic mass-regulated model, the system's diversity-maintaining capacity is finite, see Section A.6). Nevertheless, as shown by Fig. C.1A, at $m \to 0$ only one S-species survives, namely the one having the lowest growth order.

C.3.2 Invasion of an E-species into an established sub-exponential community

An invading exponential species (with $q = 1$) has a positive boundary growth rate in an established sub-exponential community (with $0 < p < 1$) if

$$\kappa\epsilon - \frac{\epsilon}{m}\,[k(m-\epsilon)^p + \kappa\epsilon] > 0. \tag{C.6}$$

After simple rearrangements, using the same approximation as in Section C.3.1, we get

$$m\frac{\kappa - km^{p-1}}{\kappa - pkm^{p-1}} > \epsilon, \tag{C.7}$$

which holds if

$$m > \left(\frac{k}{\kappa}\right)^{\frac{1}{1-p}}, \tag{C.8}$$

i.e. a minimum target replicator concentration is required for the E-species to invade an established sub-exponential community in the phenomenological model. In the mechanistic mass-regulated model, there is a critical target replicator concentration m^* for the invasion of not only an exponential, but also any sub-exponential species, see Section A.7.3.

C.3.3 Invasion of an S-species into an established exponential community

A sub-exponential species with growth order $0 < q < 1$ can invade the established exponential one (with $p = 1$) if the following holds:

$$\kappa\epsilon^q > \frac{\epsilon}{m}\left[k(m-\epsilon) + \kappa\epsilon^q\right]. \tag{C.9}$$

After simple manipulations we obtain

$$m\kappa > k\epsilon^{1-q}(m-\epsilon) + \kappa\epsilon, \tag{C.10}$$

which is always true, meaning that an S-species can always invade an E-species, enabling the coexistence of an unlimited number of S-species (while in the mechanistic mass-regulated model, the system's diversity-maintaining capacity is finite, see Section A.6).

C.3.4 Invasion of an E-species into an established exponential community

An invading exponential species (with $q = 1$) has a positive boundary growth rate in an established exponential community (with $p = 1$) if

$$\kappa\epsilon - \frac{\epsilon}{m}\left[k(m-\epsilon) + \kappa\epsilon\right] > 0. \tag{C.11}$$

After simple rearrangements, we get

$$(\kappa - k)m > (\kappa - k)\epsilon, \tag{C.12}$$

which yields the condition $m/\epsilon > 1$ if $\kappa > k$ and $m/\epsilon < 1$ if $\kappa < k$. Thus, as $\epsilon \ll m$, an E-species with a higher rate constant can invade and exclude an other E-species at any target replicator concentration m, meaning that the competitive exclusion among E-species is valid not only in the mechanistic model, but also in the phenomenological model.

References

[1] Caswell H. Matrix Population Models: Construction, Analysis, and Interpretation. Sinauer Associates Inc; 2000.

[2] The Dynamics of Physiologically Structured Populations. In: J A J Metz OD, editor. Lecture Notes in Biomathematics. vol. 68. Springer Berlin, Heidelberg; 1986. doi:https://doi.org/10.1007/978-3-662-13159-6.

[3] von Kiedrowski G. A Self-Replicating Hexadeoxynucleotide. Angewandte Chemie International Edition. 1986;25:932-5. doi:https://doi.org/10.1002/anie.198609322.

[4] von Kiedrowski G. In: Dugas H, Schmidtchen FP, editors. Minimal Replicator Theory I: Parabolic Versus Exponential Growth. vol. 3. Berlin, Heidelberg: Springer Berlin Heidelberg; 1993. p. 113-46. doi:https://doi.org/10.1007/978-3-642-78110-0_4.

[5] Zielinski WS, Orgel LE. Autocatalytic synthesis of a tetranucleotide analogue. Nature. 1987 05;327(6120):346-7. doi:https://doi.org/10.1038/327346a0.
