# Supplementary material for: The ecology and evolution of sub-exponential replicators
Source: PLoS Comput Biol. 2026 May 8;22(5):e1014247. doi: 10.1371/journal.pcbi.1014247 (PMC13155683; doi:10.1371/journal.pcbi.1014247)
Supplement: Attachment — Submitted filename: recommendation.pdf [file pcbi.1014247.s002.pdf]

PCOMPBIOL-D-25-02327: The ecology and evolution of sub-exponential replicators

Recommendation

In this work, the authors comprehensively analyse the ecological and evolutionary dynamics of sub-exponential replicators, developing a detailed analytical understanding of coexistence, invasion conditions, and stability of replicator communities. By analysing a resource-regulated model (and later showing the robustness of the results to other modelling frameworks), they derive equilibrium concentrations, growth rates, and conditions for coexistence for populations of sub-exponential and exponential replicators, and show how system parameters such as association, dissociation, replication rates, resource availability, and total mass influence community composition.

The work is clearly contextualised, the knowledge gap that it is set to fill is properly made clear, and its relevance is well defended. The main results are overall sound (but see main comment below) and, although in a simplified framework, bring theoretical clarity to the eco-evolutionary properties of populations of sub-exponential replicators. While being quite technical, the manuscript is clearly articulated, and I truly enjoyed reading it; the rich supplemental material does a great job in supporting the arguments and results of the main text.

Comment

I have one major comment, which I believe requires only a minor effort to be addressed. It concerns what I believe is a confusion in the definition of the modelling framework used throughout the work to present the major results.

The problem arises in the definition between lines 102-104. *We assume a mass-regulated setting where resource concentration is constant, and there is a constant, species-independent outflow of strands at a rate φ.* This is not the case. The model that is actually studied is akin to a turbidostat case, in which, however, the resource concentration is kept constant instead of the overall turbidity of the medium, by dynamically adjusting the outflow φ depending on the single-stranded concentrations of the different species. Indeed, Eq. (3) must hold dynamically, and not just at equilibirum, to correctly derive Eq. (A.3) in the supplemental material, and for the framework to make sense.

It is straightforward to derive this framework starting from the chemostat formulation, by imposing that $r = r^*$, where r^* is the value to which we want to keep the resource concentration constant. Imposing this on the last of the chemostat equations, Eq. (11), we get

$$0 = -r^* \sum_i c_i x_i + (\rho - r^*) D \ ,$$

Then, if we introduce the constant m and define $r^* \equiv \rho - m$, we get the sought

after functional dependence of the dilution rate from the concentration

$$\varphi \equiv D = \frac{r^*}{m} \sum_i c_i x_i \ .$$

I think correction and clarifications that put the model in the correct perspective should be made throughout the text and in the supplemental material where appropriate (starting from correcting the sentence 102-104 in the main text where φ is described as constant). Overall, this “turbidostat” model used to obtain the main results should not be treated as a simplification of the chemostat model, it should be defined for what it is, recognising that it is more transparent to be treated mathematically, and then showing that the results hold for the chemostat case, in which φ is now constant and r is free to vary (as it is already done in the manuscript). As a minor comment, in doing this modification, it could be helpful to homogenise the notation with using only φ and not D (but I don’t feel strongly about this) and to better clarify what the hatted variables are when they appear for the first time in the main text. In conclusion I believe that dropping the simplified vs. complex narrative regarding the “mass-regulated” and chemostat models, in favour of a comparison of different ecological regimes will end up strengthening the work.
