# Supplementary material for: The ecology and evolution of sub-exponential replicators
Source: PLoS Comput Biol. 2026 May 8;22(5):e1014247. doi: 10.1371/journal.pcbi.1014247 (PMC13155683; doi:10.1371/journal.pcbi.1014247)
Supplement: Attachment — Submitted filename: answerLetter.pdf [file pcbi.1014247.s003.pdf]

Budapest, 4 February 2026

Dear Dr. Grilli,

Thank you very much for your consideration of our submission, and the chance to revise and resubmit our work titled “The ecology and evolution of sub-exponential replicators”. We have rewritten the main text and the appendix based on the comments we have received. We additionally thank the Reviewers for the valuable comments, helping us improve the quality of the paper. Our point-by-point response to Referee remarks (reproduced in italics) are given below.

Thank you once again for your time and attention dedicated to our work,

Bianka Kovács, György Barabás, Géza Meszéna, Eörs Szathmáry, and András Szilágyi

Response to Reviewer 1:

The manuscript by B. Kovacs et al investigates an ecology of exponential and sub-exponential replicators. Overall, the paper is interesting and provide some new insights in the behavior such a system: (a) the rules of coexistence of a (single) exponential replicator and a set of parabolic replicators, and (b) the effect of changing the resource concentration which, somewhat counterintuitively, decreases diversity. It also recovers behavior of such dynamics systems described previously in simpler approximations of the dynamics.

Overall the manuscript is well written and the derivations in the appendix are correct. Personally, I would have liked the main technical results to be stated as formal Propositions to facilitate the exact comparison of the results with the existing literature.

We are very grateful for the supportive report. In the present manuscript, we intentionally adopted a continuous, narrative style rather than a theorem–proof format, in line with the style of the journal and its broad readership.

Regarding the behavior of the purely parabolic model, many of the results, have been described previously in the setting of Michaelis–Menten kinetics, i.e., assuming a fast equilibration of single-stranded and double stranded forms. From my reading of the present manuscript, the observed behavior is very similar. "Evolution in Systems of Ligation-Based Replicators" (Z.Phys.

web: ecolres.hun-ren.hu
eti@ecolres.hu
+36 30 462 4495
H-1121 Budapest
Konkoly-Thege Miklós út 29-33.
6. épület, 3. emelet

10.1524/zpch.2002.216.1.021) derives explicit survival conditions in equilibrium, observes the monotonic increase in diversity with increasing replicator concentration.

This is an important reference, and we thank the Reviewer for pointing it out. Indeed, Stadler et al. (2002) already demonstrated that parabolic growth does not imply unconditional coexistence. They also established the existence of concentration-dependent extinction thresholds, and showed that the number of coexisting species increases with the total replicator concentration. We now point out these prior contributions in our text (lines 78–80 and 298–301), as well as the results that go beyond them—namely, (i) that the presence of even a single exponentially growing species qualitatively alters the behavior of the system, and (ii) that diversity in sub-exponential systems depends not only on the total replicator concentration, but also on resource availability: increased resource supply will, as a rule, reduce diversity.

Prop. 2 in the same publication covers the case of invasion based on an earlier result by Hofbauer et al: It implies that the omega-limit of any trajectory starting within a subsimplex that contains an interior fixed point is that fixed point. In other words, Prop. 2 immediately implies that invasion is successful if and only if a new parabolic replicator is a survivor in the new equilibrium. (I suspect that an analogous result could be proved for the author's model.) The same publication also describes simulation results for invasion scenarios.

While we had trouble explicitly proving the global stability of an internal fixed point, our Eq (8) is essentially the analogous result mentioned by the Reviewer. In our model, invasion by a sub-exponential replicator succeeds if its intrinsic growth rate λ' is larger than the equilibrium normalized production of the system in the invader's absence: $\lambda' > \hat{\varphi}_{\text{res}}$. The equilibrium normalized production always increases as a new sub-exponential species invades a purely sub-exponential resident community, satisfying $\lambda' > \hat{\varphi}_{\text{new}} > \hat{\varphi}_{\text{res}}$ (Eq (A.36) in the Appendix). Therefore, when considering only sub-exponential species, $\lambda' > \hat{\varphi}_{\text{res}}$ implies $\lambda' > \hat{\varphi}_{\text{new}}$. This, according to Table A.1, means that the invader survives in the new equilibrium system. We now mention (lines 219–223) that for sub-exponential species, this result can be thought of as an extension to Prop. 2 in Stadler et al. (2002).

Going beyond the results for just sub-exponential species: if such a species invades a system that also includes an exponential one, then $\hat{\varphi}_{\text{new}} = \hat{\varphi}_{\text{res}}$, and thus the invasion criterion $\lambda' > \hat{\varphi}_{\text{res}}$ implies $\lambda' > \hat{\varphi}_{\text{new}}$. This means that the sub-exponential invader again survives in the new equilibrium system. That said, Eq (8) also implies that an exponential invader cannot invade a system where the normalized production has already reached the value that would be set by the given exponential invader itself, corresponding to its intrinsic growth rate. However, for a new *parabolic* replicator species, based on our numerical experience, the examined fixed points are stable, and the successful invasion of a new parabolic replicator implies its survival at the new equilibrium (and vice versa, in line with the results of Stadler et al. (2002)). To clarify this in the manuscript, we have extended the section "Invading into an established community", referring to the publication in question (lines 219–226).

As a minor note, the correspondence of a regulated flow that keeps concentrations constant and fixed flow systems was studied "Autocatalytic replication in a CSTR and constant organization" (Bull.Math.Biol 10.1007/s002850050155) showing that for small flow rates the dynamics become equivalent.

We indeed neglected discussing this in the original submission. To remediate, we have extended the description of the chemostat-type system below Eq (11), citing Happel and Stadler (1999) (lines 429–430).

In summary, I suggest to compare in more detail to the conclusions drawn in particular from the simpler Michealis–Menten type model and to focus on effects provided by the present model, if any, and effects that have not be observed already on the basis on the Michaelis-Menten approximation.

Though examining parabolic growth with respect to a different process (template-directed ligation of two fragments), Stadler et al. (2002) have indeed demonstrated some similar behaviors to those described in our manuscript. In contrast to Szathmáry and Gladkih (1989) but in line with Wills et al. (1998) and Stadler et al. (2002), we show that diversity-maintaining ability is limited even in the case of parabolic growth, and that the divergence of growth rates at low concentrations does not occur when the replication process is explicitly modeled. We also examined survival conditions, and in the Appendix (Section A.6) we explain how coexisting species can be identified from a set of parameter triplets. However, since Stadler et al. (2002) consider the build-up of the new molecules in a more detailed, different process and utilize the Michaelis–Menten approximation, the extent of the discrepancy between the two models is too large to allow for any further quantitative comparison beyond qualitative agreement. In line with this, we now refer to Stadler et al. (2002) in the Introduction (lines 78–80), and emphasize the common phenomena across these models in the Results (lines 219–223, 298–301).

Response to Reviewer 2:

In this work, the authors comprehensively analyse the ecological and evolutionary dynamics of sub-exponential replicators, developing a detailed analytical understanding of coexistence, invasion conditions, and stability of replicator communities. By analysing a resource-regulated model (and later showing the robustness of the results to other modelling frameworks), they derive equilibrium concentrations, growth rates, and conditions for coexistence for populations of sub-exponential and exponential replicators, and show how system parameters such as association, dissociation, replication rates, resource availability, and total mass influence community composition.

The work is clearly contextualised, the knowledge gap that it is set to fill is properly made clear, and its relevance is well defended. The main results are overall sound (but see main comment below) and, although in a simplified framework, bring theoretical clarity to the eco-evolutionary properties of

populations of sub-exponential replicators. While being quite technical, the manuscript is clearly articulated, and I truly enjoyed reading it; the rich supplemental material does a great job in supporting the arguments and results of the main text.

We truly appreciate the positive remarks and the relevant comment from the Referee. Our point-by-point answers to the raised issues are given below.

I have one major comment, which I believe requires only a minor effort to be addressed. It concerns what I believe is a confusion in the definition of the modelling framework used throughout the work to present the major results.

The problem arises in the definition between lines 102–104. "We assume a mass-regulated setting where resource concentration is constant, and there is a constant, species-independent outflow of strands at a rate φ." This is not the case. The model that is actually studied is akin to a turbidostat case, in which, however, the resource concentration is kept constant instead of the overall turbidity of the medium, by dynamically adjusting the outflow φ depending on the single-stranded concentrations of the different species. Indeed, Eq (3) must hold dynamically, and not just at equilibrium, to correctly derive Eq (A.3) in the supplemental material, and for the framework to make sense.

The reviewer is absolutely right, the sentence between lines 102–104 was incorrect. Actually we intended to define φ as a time-dependent quantity always determined by the current single-stranded concentrations of the different species, as formulated by Eq (3). We are grateful to the reviewer for pointing this mistake out and we have rewritten the sentence in question (lines 104–105).

It is straightforward to derive this framework starting from the chemostat formulation, by imposing that $r = r^*$, where r^* is the value to which we want to keep the resource concentration constant. Imposing this on the last of the chemostat equations, Eq (11), we get

$$0 = -r^* \sum_i c_i x_i + (\rho - r^*)D.$$

Then, if we introduce the constant m and define $r^* \equiv \rho - m$, we get the sought after functional dependence of the dilution rate from the concentration

$$\varphi \equiv D = \frac{r^*}{m} \sum_i c_i x_i.$$

We have extended the description of Eq (11) according to this remark (lines 425–428).

I think correction and clarifications that put the model in the correct perspective should be made throughout the text and in the supplemental material where appropriate (starting from correcting the sentence 102–104 in the main text where φ is described as constant). Overall, this "turbidostat" model used to obtain the main results should not be treated as a simplification of the chemostat model, it should be defined for what it is, recognising that it is more transparent to be treated mathematically, and then

web: ecolres.hun-ren.hu
eti@ecolres.hu
+36 30 462 4495
H-1121 Budapest
Konkoly-Thege Miklós út 29-33.
6. épület, 3. emelet

showing that the results hold for the chemostat case, in which φ is now constant and r is free to vary (as it is already done in the manuscript).

We have modified the introduction of the chemostat model below Eq (10) to ensure that the ``turbidostat" model does not appear as a simplification of the chemostat model but simply as a different approach (lines 414–417).

As a minor comment, in doing this modification, it could be helpful to homogenise the notation with using only φ and not D (but I don't feel strongly about this) and to better clarify what the hatted variables are when they appear for the first time in the main text. In conclusion I believe that dropping the simplified vs. complex narrative regarding the "mass-regulated" and chemostat models, in favour of a comparison of different ecological regimes will end up strengthening the work.

We have replaced D with φ everywhere, both in the main text and the supplement. We also added an explicit introduction of the hatted notation, below Eq (3) (lines 117–118).

References:

Robert Happel and Peter F. Stadler. Autocatalytic replication in a CSTR and constant organization. *Journal of Mathematical Biology*, 38(5):422–434, May 1999. ISSN 1432-1416. doi: 10.1007/s002850050155. URL https://doi.org/10.1007/s002850050155.

Bärbel M. R. Stadler, Peter F. Stadler, and Peter R. Wills. Evolution in systems of ligation-based replicators. *Zeitschrift für Physikalische Chemie*, 216(1):21, 2002. doi: doi:10.1524/zpch.2002.216.1.021. URL https://doi.org/10.1524/zpch.2002.216.1.021.

E Szathmáry and I Gladkih. Sub-exponential growth and coexistence of non-enzymatically replicating templates. *J Theor Biol*, 138(1):55–58, 05 1989.

Peter R. Wills, Stuart A. Kauffman, Barbel M. R. Stadler, and Peter F. Stadler. Selection dynamics in autocatalytic systems: Templates replicating through binary ligation. *Bulletin of Mathematical Biology*, 60(6):1073–1098, Nov 1998. ISSN 1522-9602. doi: 10.1006/S0092-8240(98)90003-9. URL https://doi.org/10.1006/S0092-8240(98)90003-9.

web: ecolres.hun-ren.hu
eti@ecolres.hu
+36 30 462 4495
H-1121 Budapest
Konkoly-Thege Miklós út 29-33.
6. épület, 3. emelet
